# Molecular characterization of humanized APOE mouse models reveals source and genotype dependent differences

**DOI:** 10.1186/s13024-026-00940-6

**Published:** 2026-03-24

**Authors:** Na Wang, Gefei Yu, Zhen Wang, Alla Alnobani, Suren Jeevaratnam, Xue Zhang, Meghan McReynolds, Yuzhou Chang, Fangfang Qi, William Tauer, Cassandra Rosenberg, Melissa Wren, Tadafumi C. Ikezu, Yuka A. Martens, Minghui Wang, Bin Zhang, Gregory W. Carter, Michael Sasner, David M. Holtzman, Junmin Peng, Long-Jun Wu, Takahisa Kanekiyo, Chia-Chen Liu, Guojun Bu

**Affiliations:** 1 Department of Neuroscience, Mayo Clinic, Jacksonville, FL USA; 2 Department of Neurology, Mayo Clinic, Rochester, MN USA; 3 Department of Genetics and Genomic Sciences, Icahn School of Medicine at Mount Sinai, New York, NY USA; 4 Departments of Structural Biology and Developmental Neurobiology, St. Jude Children’s Research Hospital, Memphis, TN USA; 5 Center for Neuroimmunology and Glial Biology, Institute of Molecular Medicine, University of Texas Health Science Center, Houston, TX USA; 6 The Jackson Laboratory, Bar Harbor, ME USA; 7 Department of Neurology, Hope Center for Neurological Disorders, Charles F. and Joanne Knight Alzheimer’s Disease Research Center, Washington University School of Medicine, St. Louis, MO USA; 8 Division of Life Science and State Key Laboratory of Nervous System Disorders, The Hong Kong University of Science and Technology, Clear Water Bay, Hong Kong, China

**Keywords:** APOE, Mouse model, Alzheimer’s disease, Lipids, Transcriptomics, Proteomics, Mass spectrometry

## Abstract

**Background:**

Humanized *APOE* targeted-replacement (TR) mice are essential tools for studying apoE isoform effects in Alzheimer’s disease (AD) and other apoE-related disorders. Despite their widespread use, existing *APOE* mouse models, generated with different gene targeting strategies, have not been directly compared in terms of apoE isoform expression, lipid profiles, and transcriptomic signatures. Such differences could impact how we interpret *APOE* genotype-related outcomes, as well as related underlying molecular mechanisms.

**Methods:**

We conducted a comprehensive molecular comparison of humanized *APOE* mouse models from three sources: Taconic Biosciences (TAC), the Cure Alzheimer’s Fund (CAF), and The Jackson Laboratory (JAX). We assessed apoE protein and transcript levels, peripheral plasma lipid composition, and bulk brain transcriptomics. ApoE isoform levels were evaluated by biochemical and proteomic measurements. Peripheral lipids, including low-density lipoprotein (LDL), high-density lipoprotein (HDL), cholesterol, and triglycerides, were also measured. We employed complementary bioinformatics analyses to evaluate brain transcriptomes and identify differentially expressed genes (DEGs) and networks based on source, *APOE* genotype, and sex.

**Results:**

We found that apoE isoforms exhibited differential levels among the three sources in the brain, liver, and plasma. Peripheral lipoproteins and lipids, including LDL, HDL, cholesterol, and triglycerides, also showed distinct concentrations in each source and genotype. Importantly, we identified distinct brain transcriptional signatures among these mouse models, which were influenced by source, *APOE* genotype, and sex. Finally, our analysis revealed specific differentially expressed genes and pathways impacted by source, genotype, and sex.

**Conclusions:**

Our findings highlight *APOE* genotype- and source-dependent variations in apoE isoform levels, lipid profiles, and molecular pathways. This study underscores the importance of consistency and caution in choosing and utilizing humanized *APOE* mouse models, offering molecular insights into key apoE-related outcomes.

**Supplementary Information:**

The online version contains supplementary material available at 10.1186/s13024-026-00940-6.

## Background

Apolipoprotein E (apoE) plays a central role in lipid transport and homeostasis within both the brain and periphery. In humans, the *APOE* gene exists in three major allelic forms - *ε2*, *ε3*, and *ε4* - which produce the corresponding protein isoforms apoE2, apoE3, and apoE4 [1–4]. These isoforms differ by single amino acid substitutions at positions 112 and 158, altering their structure and function [3, 5–9].

Alzheimer’s disease (AD), an age-related neurodegenerative disorder, is characterized by amyloid plaques, tau tangles, glial activation, and neuronal loss [5, 10–13]. The *APOE ε4* allele is the most significant genetic risk factor for late-onset AD, whereas the *APOE ε2* allele is associated with reduced risk compared to the common *ε3* allele [3, 5, 7, 14]. Beyond its well-known role in lipid transport, apoE influences key AD pathways including amyloid pathology, glial responses, synaptic function, vascular integrity, and tau-mediated neurodegeneration in an isoform-dependent manner [6, 8, 11, 15–19].

Studying apoE isoform-specific effects in the human brain is inherently challenging due to extensive genetic and environmental variability. This limitation has spurred the development of murine models to investigate the differential effects of human apoE isoforms in AD. Given that human apoE isoforms are distinct from murine apoE [16, 20–23], humanized mouse models are essential for disease-relevant research. As such, *APOE*-targeted replacement (TR) mice, initially developed to study atherosclerosis [24], have become a foundational tool for exploring isoform-specific effects of apoE in AD and related dementias [23, 25]. These models offer genetic control and experimental flexibility in a uniform background, enabling precise dissection of apoE isoform effects and related disease mechanisms.

However, despite the development of several sets of humanized *APOE*-TR mice, a direct comparison of their molecular characteristics and transcriptomic profiles is lacking. Differences in genetic engineering strategies and selection cassettes may influence apoE expression, biology, and pathobiology, contributing to variability across studies. Therefore, a systematic, head-to-head evaluation is essential to guide appropriate model selection for AD research and integration among different studies.

To address this knowledge gap, we performed, for the first time, a comprehensive molecular profiling of three widely used *APOE-*TR models sourced from Taconic Biosciences (TAC), the Cure Alzheimer’s Fund (CAF), and the Jackson Laboratory (JAX). Our study thoroughly assessed their human apoE levels, peripheral lipid profiles, and brain transcriptomes to provide essential knowledge for their optimal use as models for investigating the impact of *APOE* genotype in aging and neurodegenerative conditions.

## Method

### Animals

All animal procedures were approved by the Mayo Clinic Institutional Animal Care and Use Committee (IACUC) and followed NIH guidelines for the Care and Use of Laboratory Animals. Mice were housed in a pathogen-free, climate-controlled facility with ad libitum access to food and water. TAC, CAF and JAX mice were sourced and maintained in the animal facility at Mayo Clinic Jacksonville. Tissues were collected at 3–4 months of age for biochemical and transcriptomic analyses.

### h*APOE*-TR mouse models

Three sets of humanized *APOE*-target replacement (TR) mice in which murine *Apoe* is replaced with human *APOE* ε2/ε2, *APOE* ε3/ε3 or *APOE* ε4/ε4, were obtained from Taconic Biosciences (TAC), Cure Alzheimer’s Fund (CAF), and the Jackson Laboratory (JAX), respectively. Taconic h*APOE*-TR mice were generated through substituting exons 2–4 of the murine *Apoe* gene locus with exons 2–4 of the human *APOE* ε2, ε3, or ε4 allele, respectively, to create *APOE2*-TR, *APOE3*-TR, and *APOE4*-TR mice (*APOE2*-KI, model #1547; *APOE3*-KI, model #1548; *APOE4*-KI, model #1549). Only human *APOE* transcript can be detected in those mice after target replacement. In recent years, a new generation of ε2, ε3, or ε4 homozygous *APOE*-TR mice that possess floxed alleles for conventional or conditional deletion studies were generated by the Cure Alzheimer’s fund (CAF) (Huynh et al., 2019) for which we obtained for the current study. Further, the Jackson Laboratory created ε4/ε4 *APOE*-TR mice. Together with the resulting CRISPR/cas9 genome-edited ε3/ε3 *APOE*-TR and ε2/ε2 *APOE*-TR, these mice were also acquired for our study (*APOE4*-KI, JAX model #027894; *APOE3*-KI, JAX model #029018; *APOE2*-KI, JAX model #029017).

### Mouse tissue/plasma harvest and preparation

Before tissue and plasma extraction, mice were anaesthetized with isoflurane, followed by transcardiac perfusion using phosphate buffered saline (PBS, pH 7.4). Brain, liver and plasma samples were harvested from *n* = 3–7 mice/genotype/sex/source and stored at -80˚C. For biochemical analysis, left hemi brain was used for protein extraction and right half hemi for RNA extraction and subsequent sequencing.

### Western blotting

Brain and liver tissues were homogenized in RIPA buffer with protease and phosphatase inhibitors (Roche, Cat#11836170001; Roche, Cat#4906837001) using a Polytron homogenizer (Kinematica, Cat#PT1200E). After centrifugation (12,000 g, 15 min, 4 °C), supernatants were collected. Lysates and plasma were sonicated, re-centrifuged, and used for analysis. Protein concentration was measured via BCA assay kit (Thermofisher Scientific, Cat#23225). Equal protein amounts (or plasma volumes) were resolved by SDS-PAGE, transferred to PVDF membranes, and probed with anti-apoE (Cat#K74180B, Meridian Life Science, 1:1000), anti-β-actin (Cat#A2228, Sigma-Aldrich, 1:2000) and Serpina3n (Cat#AF4709) antibodies. Detection was performed using HRP-conjugated secondaries and quantified with the LI-COR Odyssey imaging system.

### Lipid panel measurement

Lipid profiling of unfractionated plasma, including quantification of HDL, LDL, triglycerides, and total cholesterol, was performed by the Animal Diagnostic Laboratory in the Department of Comparative Medicine at Stanford University. Biofluid analysis was conducted using the Sysmex XT-2000iV automated analyzer.

### RNA isolation, quantitative PCR and bulk RNA sequencing

Total RNA was extracted from pulverized tissue using TRIzol (ThermoFisher Scientific, Cat#15596026) and the RNeasy Mini Kit (Qiagen, Cat#74104). Genomic DNA was removed by DNase I digestion (Qiagen, Cat#79254). RNA integrity was assessed using the Agilent 4200 TapeStation with RNA ScreenTape, following the manufacturer’s protocol. All samples were processed and sequenced simultaneously to minimize batch effects.

### RNA-seq data processing and variance components analysis

Genes with counts-per-million (CPM) values greater than 0.1 in at least 5 or more samples were retained in the dataset, resulting in 21,873 genes. Gene expression was normalized using the `calcNormFactors**`** function in the edgeR R package and converted to log2(CPM) using the `voom**`** function in the limma R package [26]. To test whether any factor, such as source, *APOE* genotype, sex, or interaction of variables has a significant contribution to gene expression variance, we utilized the variancePartition R package which uses a linear mixed model to evaluate the extent of variance explained by each variable [27]. The design formula we employed in this study was (1|Sex) + (1|APOE) + (1|Source) + (1|Sex: APOE) + (1|Sex: Source) + (1|APOE: Source) + (1|Sex: APOE: Source). The results were further confirmed using the Principal Component Analysis (PCA).

### Visualization and comparison of gene expressions across group

To compare gene expression across *APOE* genotypes, sex and sources, we visualized voom-transformed log2 counts per million (log2CPM) values using violin plots. Each data point represents an individual mouse, with point shape indicating sex (female: filled circle; male: open circle) and point color indicating *APOE* genotype (E2: green; E3: yellow; E4: red). For each source, pairwise comparisons between *APOE* genotypes were performed using the Wilcoxon rank-sum test. Adjusted p-values were computed using the Benjamini-Hochberg method, and comparisons with adjusted *p* < 0.05 were annotated as “***”.

### Differential gene expression analysis

Differential expression analyses were conducted using the R package limma. P values were adjusted for multiple testing using the Benjamini-Hochberg (BH) method, and differentially expressed genes (DEGs) were defined with BH-adjusted p value < 0.05 and |log2 (FC)| ≥ log2(1.2). Volcano plots were generated using the EnhancedVolcano R package, with gene symbols containing “Gm” excluded from labeling for clarity [28]. Venn diagrams illustrating the overlap of DEGs across different comparisons were produced using the ggvenn package [29]. Gene Ontology (GO) enrichment analysis of DEGs was performed using the hypergeometric test with the GOtest package using the Molecular Signatures Database (MSigDB). Additionally, unsupervised hierarchical clustered heatmaps were generated using z-scaled expression values of top DEGs and the `Heatmap**`** function with the Euclidean distance method in the ComplexHeatmap R package [30].

### Weighted gene co-expression network analysis (WGCNA)

We used the WGCNA R package to construct the signed-hybrid co-expression network for all samples [31]. The soft-thresholding power (β = 6) was selected in a data-driven manner following standard WGCNA criteria and demonstrates appropriate adherence to scale-free topology. The minimum module sizes were set to 20 and we used mergeCutHeight = 0.3 to merge modules with correlation coefficients greater than 0.7 into one. Module eigengenes (MEs), calculated by the first principal component of each module, were used to test the correlation of each module with the traits of interest: Sources, Sex, and *APOE* genotype. Module eigengene expression in each group was illustrated using boxplots, with each point representing an individual mouse. Gene ontology enrichment analysis was performed for each module using the clusterProfiler R package. For selected modules, the top 10 hub genes-ranked by intramodular connectivity (kME)-were visualized using the igraph package (10.32614/cran.package.igraph).

### ApoE quantification from plasma, brain, and liver samples by LC-MS/MS

ApoE protein levels were quantified from mouse plasma, brain, and liver samples using liquid chromatography–tandem mass spectrometry (LC-MS/MS).

Sample preparation: Plasma samples from various mouse models were diluted to a final protein concentration of 1 µg/µL in SDS lysis buffer containing 2% SDS, 50 mM HEPES (pH 8.5), and protease inhibitors (1×). Proteins were denatured by heating at 95 °C for 10 min. Brain and liver tissues were weighed and lysed in urea lysis buffer (100 µL/10 mg tissue) composed of 8.8 M urea, 50 mM HEPES (pH 8.5), 0.5% sodium deoxycholate, protease inhibitors (1×), and 20% (v/v) glass beads. Protein extraction was performed by beads beating in the urea lysis buffer as previously described [32]. A total of 10 µg protein from each sample across plasma, brain or liver was reduced with 5 mM dithiothreitol (DTT) for 30 min, followed by alkylation with 20 mM iodoacetamide (IAA) in the dark for 30 min. The alkylation reaction was quenched with 20 mM DTT for 30 min. Protein cleanup and tryptic digestion were performed using the single-pot, solid-phase-enhanced sample preparation (SP3) method [33]. The resulting peptides were dried using a SpeedVac and resuspended in 5% formic acid (FA) prior to LC-MS/MS analysis.

LC-MS/MS analysis: Peptides were analyzed on a Q-Exactive HF Orbitrap mass spectrometer (Thermo Fisher Scientific) using a 15-minute nano-LC gradient from 16% to 40% buffer B (buffer A: 0.2% FA, 3% DMSO; buffer B: buffer A with 65% acetonitrile). Data acquisition was performed using a hybrid method combining top-3 data-dependent acquisition (DDA) and parallel reaction monitoring (PRM) [34, 35]. DDA MS1 parameters included: 30,000 resolution, 400–1000 *m/z* scan range, 1 × 10^6^ AGC, and 50 ms maximal ion time. DDA MS2 scans were acquired at 15,000 resolution with 1 × 10^5^ AGC, 20 ms maximal ion time, 1.0 *m/z* isolation window with 0.2 *m/z* offset, 30% HCD normalized collision energy (NCE), and 10 s dynamic exclusion. PRM scans were acquired at 15,000 resolution with 1 × 10^5^ AGC, 120 ms maximum ion time, 2.0 *m/z* isolation window with 0.2 *m/z* offset, 30% NCE. The following *m/z* values were targeted: 749.4046, 810.9025, 865.9258, 554.7715, 503.2374, 611.7527, and 474.7669, corresponding to peptides AATVGSLAGQPLQER, VQAAVGTSAAPVPSDNH, SELEEQLTPVAEETR, CLAVYQAGAR, LGADMEDVR, LGADMEDVCGR, and LAVYQAGAR, respectively.

Data analysis: Raw MS data were searched using FragPipe v22.0 against a combined mouse proteome database compiled from Swiss-Prot, TrEMBL (UniProt), and UCSC (59,423 entries), with human *APOE* isoforms (E2, E3, and E4) manually appended. Following spectral matching, a spectral library was generated in FragPipe for Skyline precursor extraction, and apoE precursor ion intensities were extracted in Skyline. ApoE quantification was performed by multiplying average of 3 apoE shared peptide precursor intensities with peptide-wise mean-centered intensities [36]. ApoE shared peptides across all isoforms are: AATVGSLAGQPLQER, VQAAVGTSAAPVPSDNH, and SELEEQLTPVAEETR. The peptide intensities of CLAVYQAGAR, LGADMEDVR, LGADMEDVCGR and LAVYQAGAR were used to confirm *APOE* genotypes. Other protein intensities derived from DDA scans quantified by FragPipe were used to normalize the loading bias.

### Statistical analysis

Statistical methods for RNA-sequencing analysis are detailed in related sections or figure legends. Statistical analyses for biochemical studies were performed using GraphPad Prism 10. Methods for each analysis are detailed in the figure legends. Statistical significance was determined using one-way ANOVA or two-way ANOVA followed by Tukey’s multiple comparisons test to assess differences between genotypes and sources for biochemical measurements. Data are presented as mean ± SEM, with n values indicated in the figure legends. N.S., not significant. Significance was defined as *p < 0.05, **p < 0.01, ***p < 0.001, ****p < 0.0001.

## Result

### Gene-targeting strategies of humanized *APOE*-targeted replacement (TR) mouse models from three sources

**Fig. 1:**
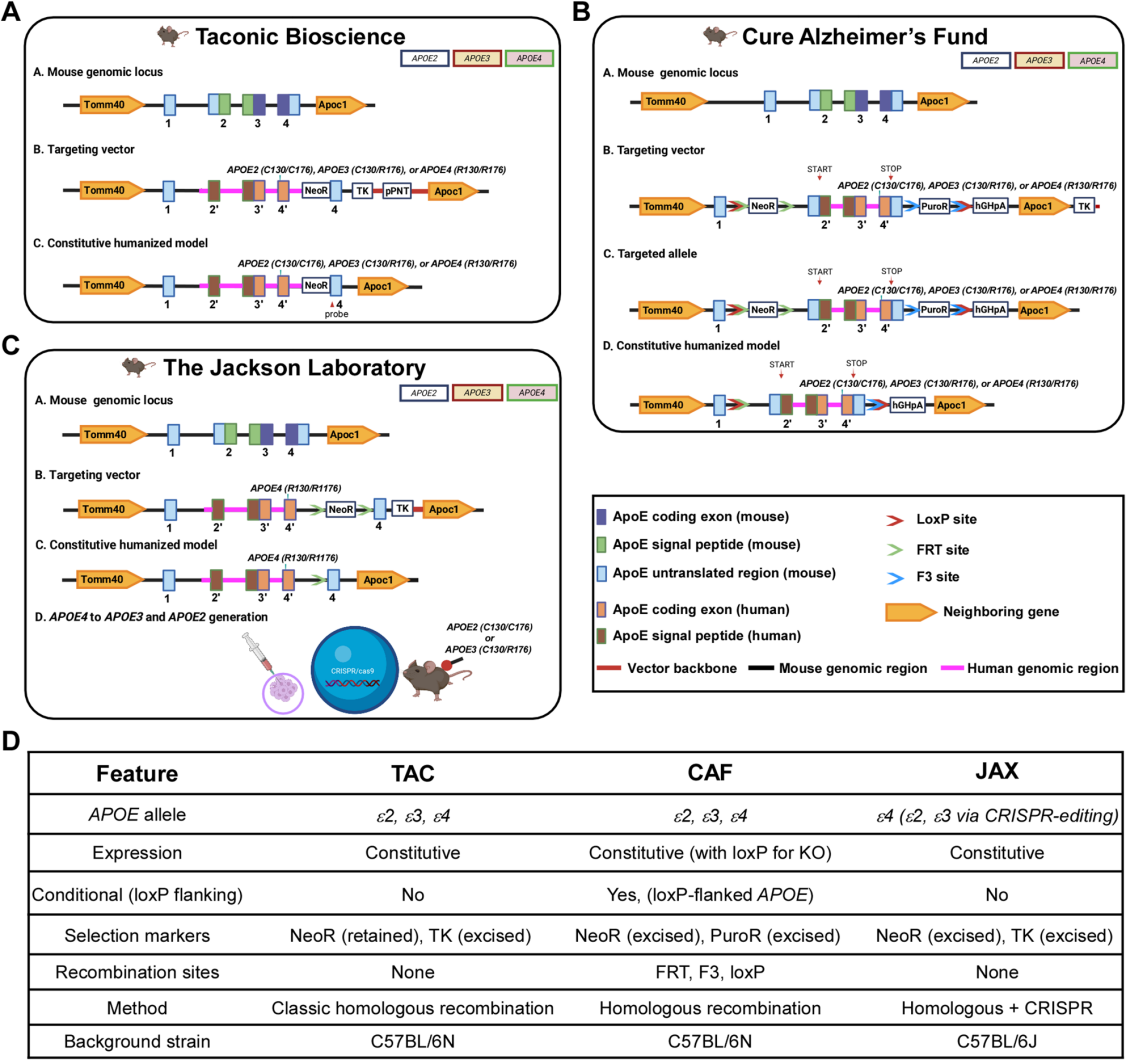
Gene targeting strategies for three sources of humanized *APOE*-targeted replacement (TR) mice. **(A-C)** Model design strategies for Taconic Biosciences (TAC) (**A**), Cure Alzheimer’s Fund (CAF) **(B)**, and the Jackson Laboratory (JAX) *APOE*-TR mice **(C)**. The targeting strategies involve the genomic structure of the mouse *Apoe* gene encompassing exons 1–4, with targeting vectors designed to replace mouse *Apoe* exons 2–4 with corresponding coding sequences from human *APOE* ε2, ε3, or ε4 alleles (2’, 3’, 4’) **(A-C)**. The mouse exon 4 probe (*Probe*,* indicated by red arrow*) was used to detect the targeted allele in TAC mice **(A)**. Notably, the human *APOE* sequence in CAF mice is flanked by loxP sites to enable cre-mediated conditional deletion of the *APOE* gene; and a polyadenylation signal (hGHpA: human Growth Hormone polyadenylation signal) has been inserted to the 3′ of the genes (downstream of the distal loxP sites) in order to prevent transcriptional read-through **(B)**. The JAX mice were originally generated by targeted replacement with the human *APOE* ε4 allele, whereas the *APOE3*-TR and *APOE2*-TR mice were generated subsequently by CRISPR-mediated gene editing of the *APOE4*-TR mice **(C)**. Selection markers vary among the models: TAC and JAX mice incorporate positive and negative selection markers including Neomycin resistance (NeoR) and Thymidine kinase (TK), respectively (**A**,** C**); whereas CAF mice utilize NeoR and Puromycin resistance (PuroR) cassettes flanked by FRT and F3 recombination sites positioned downstream and upstream of the loxP sites **(B)**. In all models, the human *APOE* gene is expressed under the control of the endogenous mouse *Apoe* promoter, ensuring physiological regulation. Importantly, TAC mice retain NeoR selection cassettes **(A)**, while these markers were removed in CAF and JAX models **(B**,** C)**. **(D)** Summary table detailing the differences in gene targeting strategies among the three sources of humanized APOE-TR mouse models

Three major sources provide humanized *APOE*-targeted replacement (*APOE*-TR) mice: Taconic Biosciences (TAC) [24, 37, 38], the Cure Alzheimer’s Fund (CAF) [17, 25, 39], and the Jackson Laboratory (JAX) [40–42]. All these models replace mouse *Apoe* exons 2–4 with the corresponding human coding sequences (*ε2*, *ε3*, or *ε4*) under control of the mouse endogenous *Apoe* promoter to ensure physiological expression [24, 25, 40]. TAC *APOE*-TR mice (C57BL/6N background) retain the neomycin (NeoR) selection cassette and express human *APOE* constitutively [24, 37, 38] (Fig. 1A, D). CAF *APOE*-TR mice (also C57BL/6N) incorporate both NeoR and puromycin (PuroR) cassettes flanked by recombination sites (FRT, F3), which are removed in the final line, and uniquely include loxP sites flanking the human *APOE* sequence, enabling cre-mediated conditional deletion [25] (Fig. 1B, D). JAX *APOE*-TR mice differ in substrain genetic background (C57BL/6J) and origin, with ε2 and ε3 alleles generated by CRISPR editing of an initial ε4 knock-in after selection cassettes had been removed [40–42] (Fig. 1C, D). For clarity, the distinct gene-targeting strategies used to generate the *APOE*-TR mouse models are summarized in the table presented in Fig. 1D.

### Comparative characterization of humanized *APOE*-TR mice from three sources

**Fig. 2:**
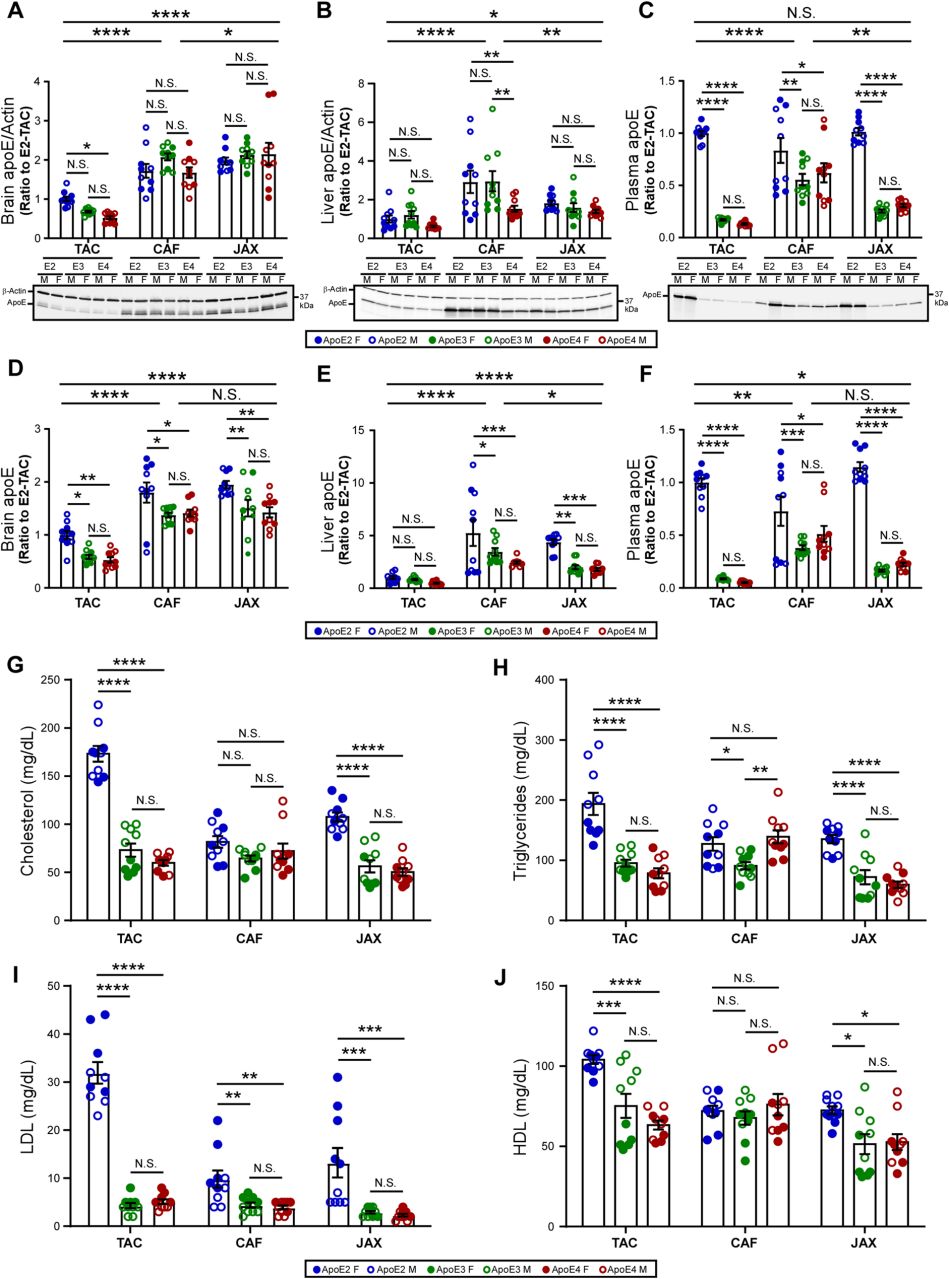
Molecular characterization of humanized *APOE*-TR mice. **(A-C)** ApoE levels measured by western blotting (WB). *n* = 3–5 mice/source/*APOE* genotype/sex. **(D-F)** ApoE levels measured by mass spectrometry. *n* = 3–5 mice/source/*APOE* genotype/sex. **(G-J)** Measurements of cholesterol, triglycerides, LDL, and HDL concentration (mg/dL) by lipid panel. *n* = 5 mice/source/*APOE* genotype/sex. Two-way ANOVA or one-way ANOVA with Tukey multiple comparisons test was used for comparison of *APOE* genotype within each source and comparison of sources. Data are presented as mean ± SEM. N.S., not significant. Significance was defined as *p < 0.05, **p < 0.01, ***p < 0.001, ****p < 0.0001

Given that *APOE*-TR mice from TAC, CAF, and JAX were generated using different gene-targeting strategies (Fig. 1A-D), we first comprehensively examined *APOE* level in the brain, liver, and plasma across different *APOE* genotypes and sources. Western blotting analysis revealed an apoE isoform-dependent pattern in TAC *APOE*-TR mouse brains (apoE2 > apoE3 > apoE4), a trend notably absent in the brains of *APOE*-TR mice sourced from CAF or JAX (Fig. 2A). However, subsequent mass spectrometry (MS) analysis indicated the levels of brain apoE2 in general trended higher than apoE3 and apoE4 across all three sources. Notably, the overall levels of brain apoE are lower in the TAC mice than those in CAF and JAX mice across apoE isoforms (Fig. 2A, D). In the liver, isoform-specific differences were only detected in CAF by western blotting (Fig. 2B), although an apoE2 ≥ apoE3 ≥ apoE4 trend across sources appeared when analyzed by mass spectrometry (Fig. 2E). The overall levels of liver apoE isoforms in the TAC mice are again lower than those in the CAF and JAX mice across apoE isoforms (Fig. 2B, E). Plasma apoE levels, across both detection methods, showed consistent trends in TAC and JAX mice, with markedly higher in *APOE2* mice than in *APOE3* or *APOE4* counterparts (Fig. 2C, F), suggesting a hyperlipoproteinemia phenotype in *APOE2* mice.

Interestingly, JAX mice showed the lowest *APOE* transcript levels (Figure S1A) compared to CAF and TAC mice, despite having relatively higher protein levels than TAC mice. This discrepancy might suggest differences in translation efficiency, RNA stability, and/or protein turnover. Further transcriptomic analysis revealed that the expression levels of *APOE*-related genes *Lrp1* and *Lrp6* were lower in JAX mice, while *Clu* (*ApoJ*) was elevated (Figure S1B). Notably, members of the *Serpina3* gene family, including *Serpina3n*, *Serpina3m*, and *Serpina3k*, were significantly upregulated in TAC *APOE4* mice (Figure S1C), consistent with previous findings [43]). This specific pattern was not observed in the CAF or JAX mice.

To investigate the impact of *APOE* isoform on peripheral lipids, we assessed unfractionated plasma lipid profiles, including cholesterol, triglycerides, low-density lipoprotein (LDL), and high-density lipoprotein (HDL), through the Animal Diagnostic Laboratory at Stanford University (Fig. 2G-J). Cholesterol and triglyceride levels exhibited consistent isoform-dependent differences within the TAC and JAX mice. Specifically, *APOE2* mice from both sources displayed significantly higher concentrations of cholesterol and triglyceride compared to *APOE3* and *APOE4* mice, where no differences were observed between *APOE3* and *APOE4* mice (Fig. 2G, H). In CAF mice, no significant differences in cholesterol levels were detected among isoforms; however, triglyceride levels did vary by *APOE* genotype (Fig. 2H). LDL levels followed a similar isoform-dependent pattern across all three sources, with significantly higher concentrations in *APOE2* mice. No differences were observed between *APOE3* and *APOE4* genotypes within any source (Fig. 2I). HDL levels were also elevated in TAC and JAX *APOE2* mice but showed no significant difference between *APOE3* and *APOE4* animals from those sources. Notably, no isoform-dependent differences in HDL levels were detected in CAF mice (Fig. 2J). These findings collectively suggest that *APOE* genotype exerts source-dependent effects on peripheral lipid metabolism, with *APOE2* generally associated with elevated lipid levels, particularly in the TAC and JAX mice.

### Transcriptomic profiling of brain samples from *APOE*-TR mice with different source, *APOE* genotype, and sex

**Fig. 3:**
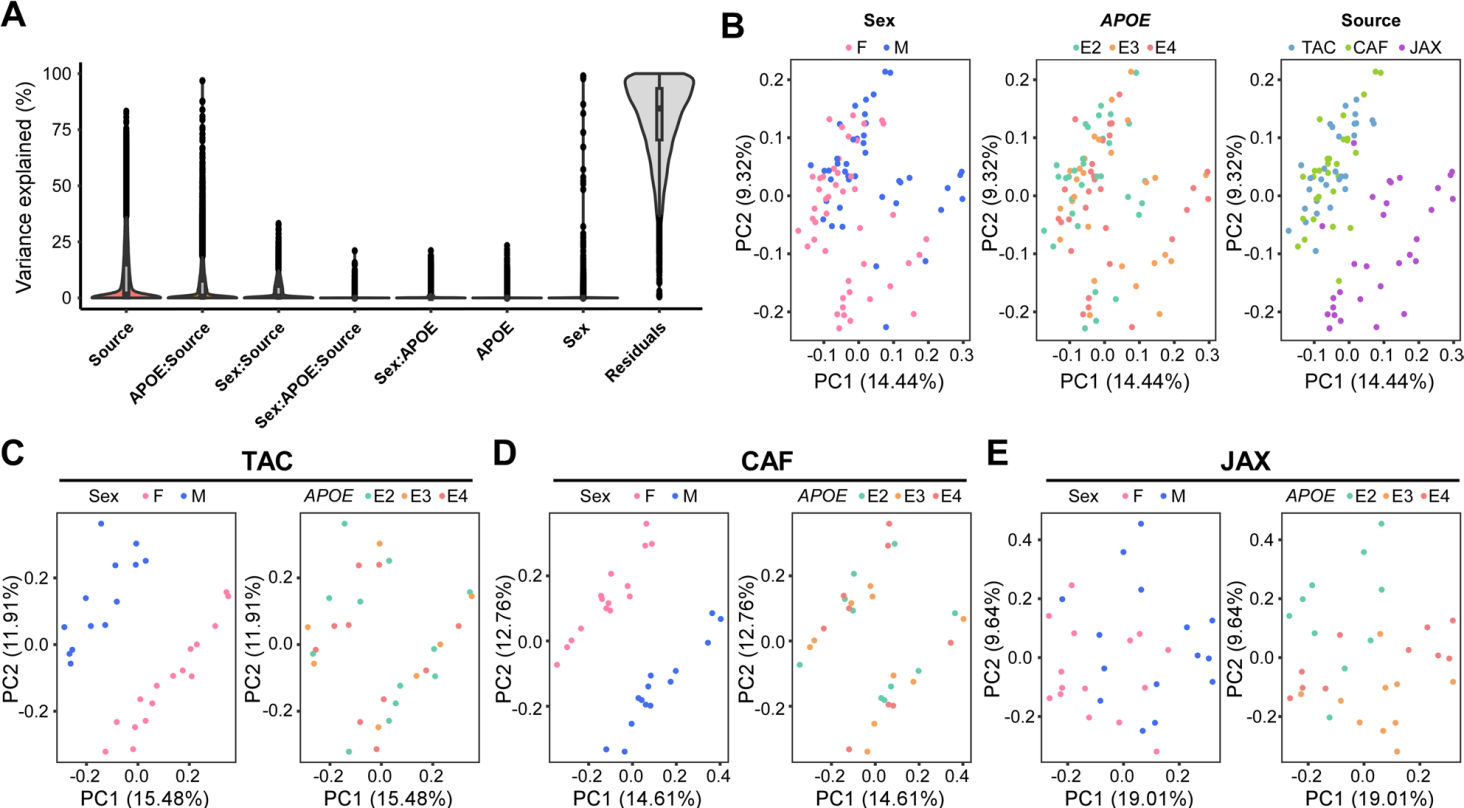
Global characterization of brain transcriptomes of *APOE*-TR mice. **(A)** The effect of source, *APOE* genotype, sex and their interactions on the variation of brain gene expression in the study cohort (*n* = 3–7 mice/genotype/source/sex). **(B)** Sample-to-sample variation among brain transcriptomes revealed by principal component analysis (PCA). Each dot represents a sample, colored by source, *APOE* genotype, and sex, respectively. **(C-E)** Sex effect on brain transcriptomes revealed by PCA within each source. VariancePartition R package was utilized to evaluate the extent of variance explained by each variable; The results were further confirmed using the PCA (see details in method section)

Given the widespread use of *APOE*-TR mice in neurodegenerative disease research, we conducted a comprehensive transcriptomic analysis on pulverized brain tissues from 3-4-month-old mice (3–7 mice per genotype/sex/source; Table S1) representing three different sources: TAC, CAF, and JAX. Our primary objective was to determine how source, *APOE* genotype, and sex contribute to global gene expression differences in the brain. Principal variance component analysis (PVCA) revealed that source was the primary driver of transcriptomic variation (Fig. 3A). Supporting this, principal component analysis (PCA) demonstrated clearly a distinct separation of JAX samples from those of TAC and CAF, whereas *APOE* genotype exhibited minimal separation across all groups (Fig. 3B). Notably, male and female samples clustered separately, indicating a measurable sex-specific effect on the brain transcriptome. We further investigated genotype and sex effects within each source. PCA demonstrated clear sex-based separation in both TAC and CAF cohorts (Fig. 3C, D), while JAX mice displayed minimal sex-based clustering (Fig. 3E). Consistent with the global analysis, *APOE* genotype did not result in distinct separation within any of the three sources (Fig. 3C-E), suggesting that, at this young adult age, genotype alone has a relatively modest effect on bulk brain transcriptomic profiles.

### Identification of differentially expressed genes (DEGs) and enriched pathways in the brains of *APOE*-TR mice across three sources

**Fig. 4:**
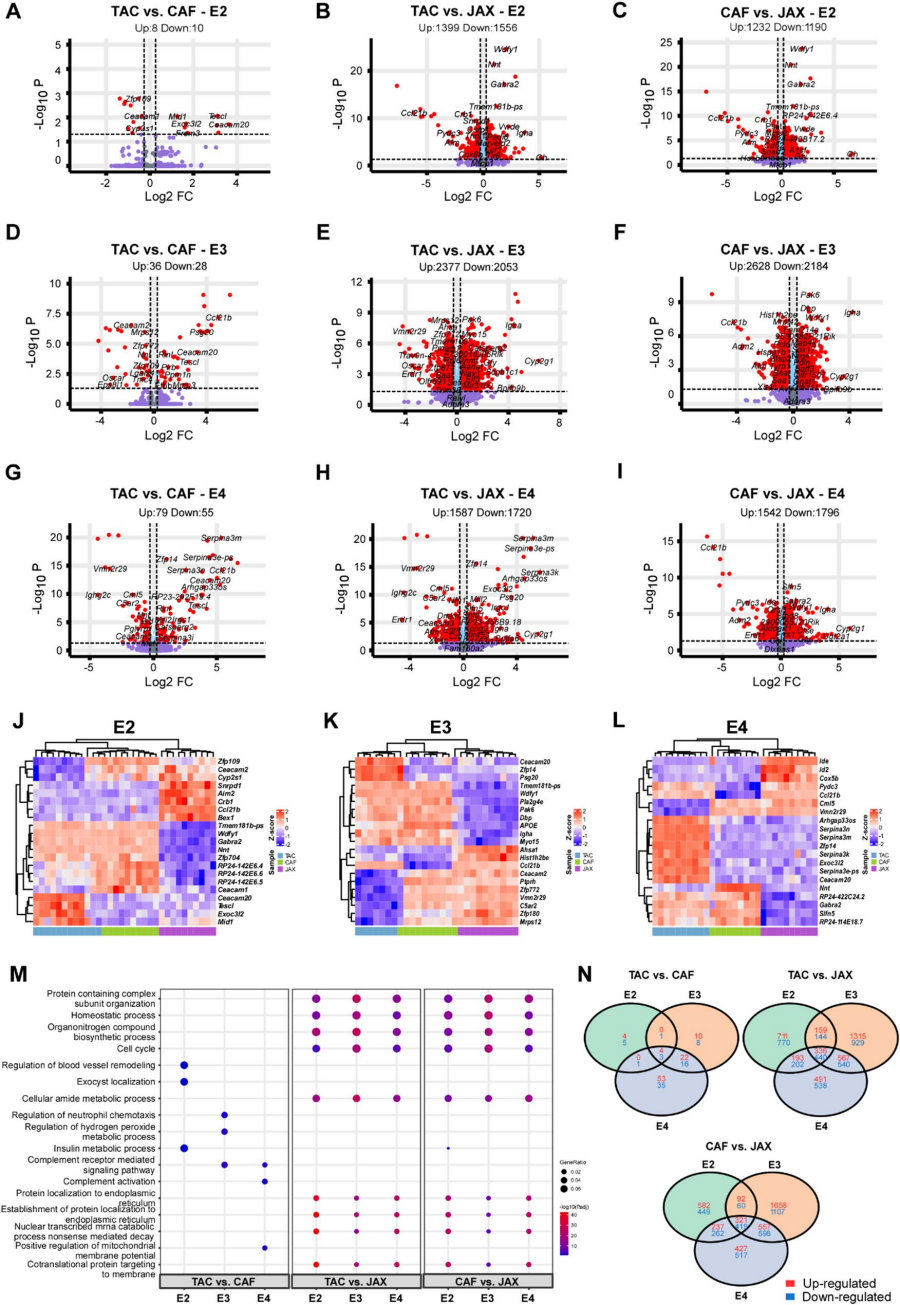
Differential gene expression and pathway analyses of the mouse brain transcriptomes affected by source in each *APOE* genotype. **(A-I)** Volcano plots of DEGs identified among the sources in each *APOE* genotype (A-C, *APOE2*; D-F, *APOE3*; G-I, *APOE4*). The red dots on the left denote downregulated differentially expressed genes (DEGs), and the red dots on the right denote upregulated DEGs in each comparison. P values were adjusted for multiple testing using the Benjamini-Hochberg (BH) method, and DEGs were defined with BH-adjusted p value < 0.05 and |log2 (FC)| $\:>$ log2(1.2) (see details in method section). The light blue dots denote genes with significant p values but |fold change| $\:\le\:$1.2, and the purple dots denote genes that did not meet either the p value or the fold change threshold. **(J-L)** Hierarchical clustering of the top DEGs affected by source in each genotype. Each row corresponds to one gene and each column corresponds to one sample. **(M)** Top Gene Ontology (GO) pathways enriched by DEGs affected by source in each genotype. **(N)** Venn diagrams illustrating the overlap of DEGs across different comparisons

We then analyzed differentially expressed genes (DEGs) associated with source, *APOE* genotype, and sex (Table S2). To begin, we specifically examined the impact of mouse model sources on gene expression profiles and pathway enrichment within each *APOE* genotype (Fig. 4A-N).

In mice carrying the *APOE2* genotype, we identified a notable number of DEGs across sources: 8 upregulated and 10 downregulated DEGs in the TAC vs. CAF comparison; 1,399 upregulated and 1,556 downregulated DEGs in TAC vs. JAX; and 1,232 upregulated and 1,190 downregulated DEGs in CAF vs. JAX (Fig. 4A-C, N). Among these, *Zfp109*, *Ceacam2*, and *Cyp2s1* were downregulated in TAC *APOE2* mice compared to both CAF and JAX mice, whereas *Ceacam20*, *Tescl*, *Exoc3l2*, and *Mid1* were upregulated (Fig. 4J). In contrast, *Tmem181b-ps*, *Wdfy1*, *Gabra2*, *Nnt*, *Zfp704*, and the non-coding transcripts *RP24-142E6.4*, *RP24-142E6.5*, *RP24-142E6.6*, and *Ceacam1* were downregulated in JAX *APOE2* mice compared to TAC and CAF mice, while *Snrpdf*, *Aim2*, *Crb1*, *Ccl21b*, and *Bax1* were upregulated (Fig. 4J). Pathway enrichment analysis revealed that DEGs in TAC vs. CAF *APOE2* mice were associated with blood vessel remodeling, exocyst localization, and insulin metabolic processes (Fig. 4M). In contrast, DEGs in TAC vs. JAX and CAF vs. JAX *APOE2* comparisons were predominantly enriched in pathways related to cellular metabolism, including protein localization, organonitrogen compound biosynthesis, cell cycle regulation, amide metabolism, mRNA catabolism, and co-translational protein targeting to membranes (Fig. 4M).

For mice with the *APOE3* genotype, we identified 36 upregulated and 28 downregulated DEGs in the TAC vs. CAF comparison; 2,377 upregulated and 2,053 downregulated DEGs in TAC vs. JAX; and 2,628 upregulated and 2,184 downregulated DEGs in CAF vs. JAX (Fig. 4D-F, N). Among these, Ceacam20, Zfp14, and Psg20 were upregulated, whereas *Ceacam2*, *Ptprh*, *Zfp772*, *Vmn2r29*, *C5ar2*, *Zfp180*, and *Mrps12* were downregulated in TAC *APOE3* mice relative to both CAF and JAX *APOE3* mice (Fig. 4K). In JAX *APOE3* mice, *Tmem181b-ps*, *Wdfy1*, *Pla2g4e*, *Pak6*, *Dbp*, *APOE*, *Igha*, and *Myo15* were downregulated, while *Ahsa1*, *Hist1h2be*, and *Ccl21b* were upregulated compared to TAC and CAF *APOE3* mice (Fig. 4K). Pathway enrichment analysis showed that DEGs in TAC vs. CAF *APOE3* mice were primarily associated with immune-related processes, including the complement receptor–mediated signaling pathway, neutrophil chemotaxis regulation, and hydrogen peroxide metabolic regulation (Fig. 4M). Similar to the *APOE2* group, DEGs in both TAC vs. JAX and CAF vs. JAX *APOE3* comparisons were enriched in cellular metabolism–related pathways, though with lower statistical significance (Fig. 4M).

In *APOE4* mice, we identified 79 upregulated and 55 downregulated DEGs in TAC vs. CAF, 1,587 upregulated and 1,720 downregulated DEGs in TAC vs. JAX, and 1,542 upregulated and 1,796 downregulated DEGs in CAF vs. JAX (Fig. 4G-I, N). In JAX *APOE4* mice, genes such as *Ide*, *Id2*, and *Cox5b* were significantly downregulated, as were *Nnt*, *RP24-422C24.2*, *Gabra2*, *Slfn5*, and *RP24-114E18.7* (Fig. 4L). Notably, *Serpina3* family genes, including *Serpina3n*, *Serpina3m*, *Serpina3k*, and *Serpina3e-ps*, along with *Arhgap33os*, *Zfp14*, *Exoc312*, and *Ceacam20*, were robustly upregulated in TAC *APOE4* mice compared to CAF and JAX *APOE4* mice. Conversely, *Cml5* and *Vmn2r29* were downregulated in TAC *APOE4* mice (Fig. 4L). This increased expression of *Serpina3* genes in TAC *APOE4* mice aligns with prior findings from our group [43]. In contrast, *Pydc3* and *Ccl21b* were downregulated in CAF *APOE4* mice relative to both TAC and JAX *APOE4* mice (Fig. 4L). Pathway enrichment analyses revealed that DEGs in the TAC vs. CAF *APOE4* comparison were enriched in complement cascade-related and mitochondrial membrane-associated pathways (Fig. 4M). Similar to the *APOE2* and *APOE3* genotypes, DEGs from both TAC vs. JAX and CAF vs. JAX *APOE4* comparisons were predominantly enriched in cellular metabolism pathways (Fig. 4M).

### Identification of *APOE* genotype-related DEGs and enriched pathways across three sources in the brains of *APOE*-TR mice

To further unravel the impact of *APOE* genotype on brain transcriptomes, we identified DEGs and their associated biological pathways among *APOE2*,* APOE3*, and *APOE4* genotypes within each of the three humanized *APOE*-TR mouse sources (TAC, CAF, and JAX).

**Fig. 5:**
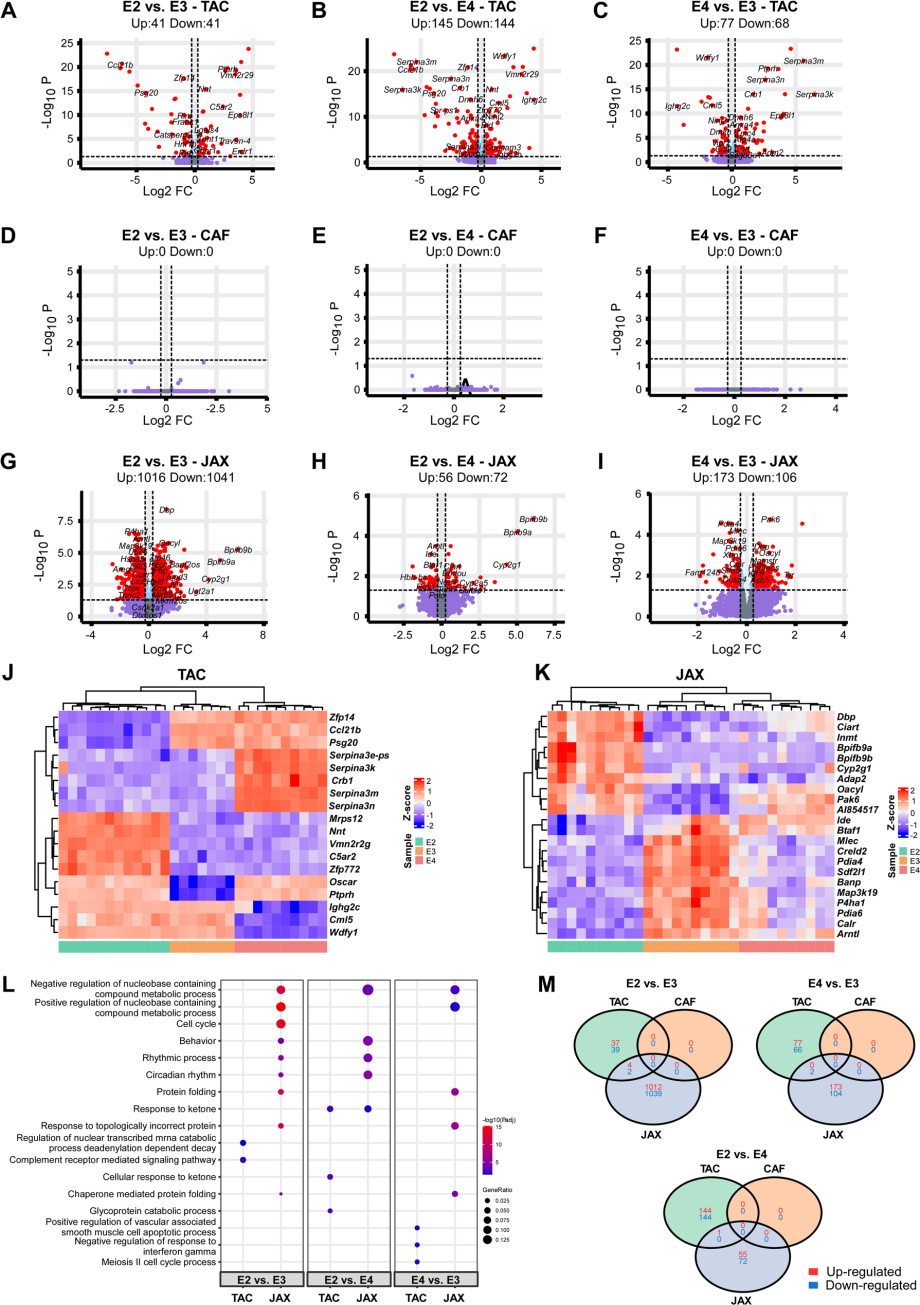
Differential gene expression and pathway analyses of the mouse brain transcriptomes affected by *APOE* genotype in each source. **(A-I)** Volcano plots of DEGs identified among the *APOE* genotypes in individual sources (**A-C**, Taconic Bioscience; **D-F**, Cure Alzheimer’s Fund; **G-I**, The Jackson laboratory). The red dots on the left denote downregulated DEGs, and the red dots on the right denote upregulated DEGs in each comparison. P values were adjusted for multiple testing using the BH method, and DEGs were defined with BH-adjusted p value < 0.05 and |log2 (FC)| $\:>\:$log2(1.2). The light blue dots denote genes with significant p values but |fold change| $\:\le\:\:$1.2, and the purple dots denote genes that did not meet either the p value or the fold change threshold. **(J-K)** Hierarchical clustering of the top DEGs affected by *APOE* genotype. Each row corresponds to one gene and each column corresponds to one sample. **(L)** Top GO pathways enriched by DEGs affected by *APOE* genotype in each source. **(M)** Venn diagrams illustrating the overlap of DEGs across different comparisons

We specifically examined how *APOE* genotype influenced DEGs and pathway enrichment within each *APOE*-TR mouse source (Fig. 5A-M). In TAC mice, comparisons revealed 41 upregulated and 41 downregulated DEGs in *APOE2* vs. *APOE3*; 145 upregulated and 144 downregulated DEGs in *APOE2* vs. *APOE4*; and 77 upregulated and 68 downregulated DEGs in *APOE4* vs. *APOE3* (Fig. 5A-C, M). Hierarchical clustering of top DEGs across these comparisons demonstrated clear separation by *APOE* genotype (Fig. 5J). As anticipated, several members of the Serpina3 gene family, including *Serpina3n*, *Serpina3m*, *Serpina3k*, and *Serpina3e-ps*, as well as *Crb1*, were upregulated in *APOE4* compared to *APOE2* and *APOE3*. In contrast, *Ighg2c*, *Cml5*, and *Wdfy1* were downregulated in *APOE4* mice (Fig. 5J). Genes such as *Zfp14*, *Ccl21b*, and *Psg20* were downregulated in *APOE2* mice, while *Mrps12*, *Nnt*, *Vmn2r2g*, *C5ar2*, and *Zfp772* were upregulated relative to both *APOE3* and *APOE4* (Fig. 5J). Additionally, *Oscar* and *Ptprh* were downregulated in *APOE3* mice compared to the other two genotypes. Pathway enrichment analysis of DEGs revealed distinct biological processes associated with each comparison: DEGs from *APOE2* vs. *APOE3* were enriched in complement receptor-mediated signaling and nuclear-transcribed mRNA catabolic processes. *APOE2* vs. *APOE4* DEGs were associated with ketone response and glycoprotein catabolic pathways. In *APOE4* vs. *APOE3*, enriched pathways included vascular smooth muscle cell apoptosis, interferon gamma response, and cell cycle regulation (Fig. 5L).

In JAX mice, we identified substantial *APOE* genotype-dependent transcriptomic differences. Specifically, there were 1016 upregulated and 1041 downregulated DEGs in *APOE2* vs. *APOE3*, 56 upregulated and 72 downregulated DEGs in *APOE2* vs. *APOE4*, and 173 upregulated and 106 downregulated DEGs in *APOE4* vs. *APOE3* (Fig. 5G-I, M). Hierarchical clustering of the top DEGs revealed clear segregation by *APOE* genotype (Fig. 5K). Several genes were uniquely regulated in *APOE2* mice compared to *APOE3* and *APOE4*. Upregulated genes in *APOE2* included *Dbp*, *Ciart*, *Inmt*, *Bpifb9a*, *Bpifp9b*, *Cyp2g1*, *Adap2*, *Oacyl*, *Pak6*, and *Al854517*. Conversely, genes such as *Ide*, *Btaf1*, *Mlec*, *Creld2*, *Pdia4*, *Sdf2l1*, *Banp*, *Map3k19*, *P4ha1*, *Pdia6*, *Calr*, and *Arntl* were downregulated in *APOE2* but showed increased expression in *APOE3* (Fig. 5K). Pathway enrichment analysis revealed that DEGs from *APOE2* vs. *APOE3* were primarily associated with nucleobase-containing compound metabolism, cell cycle regulation, and protein folding. DEGs from *APOE2* vs. *APOE4* were enriched in pathways related to cellular behavior, circadian rhythm, and ketone response. Similarly, *APOE4* vs. *APOE3* DEGs were enriched in pathways involved in nucleobase metabolism and protein folding (Fig. 5L). In contrast to TAC and JAX models, no significant DEGs were detected among *APOE* genotypes in CAF mice using the same analytical approach (Fig. 5D-F).

### Identification of sex-related DEGs and enriched pathways across three sources of *APOE*-TR mice

**Fig. 6:**
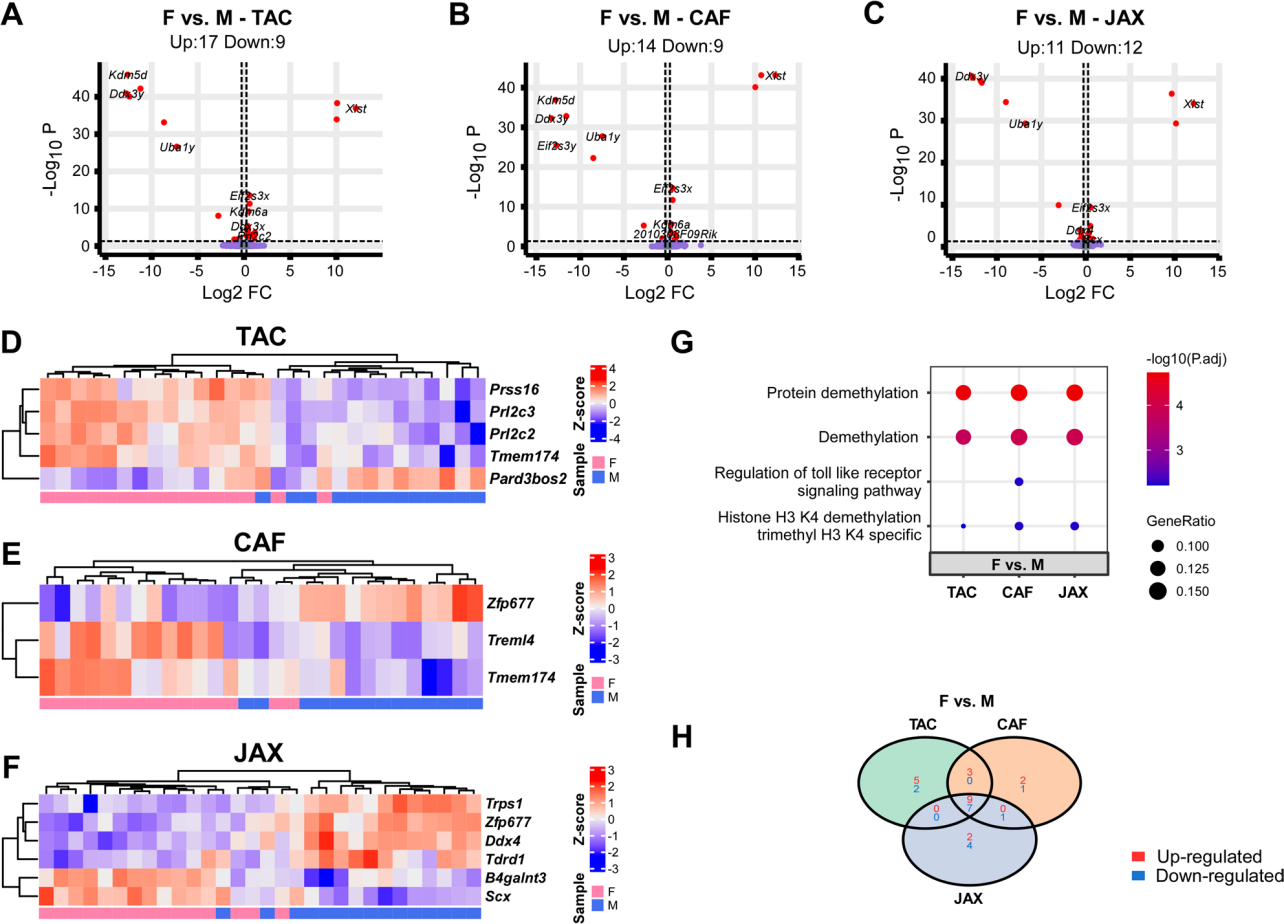
Differential gene expression and pathway analyses of the mouse brain transcriptomes affected by sex in each source. **(A-C)** Volcano plots of DEGs identified between the female and male in each source (**A**, TAC; **B**, CAF; **C**, JAX). The red dots on the left denote downregulated DEGs, and the red dots on the right denote upregulated DEGs in each comparison. P values were adjusted for multiple testing using the BH method, and DEGs were defined with BH-adjusted p value < 0.05 and |log2 (FC)| $\:>$ log2(1.2). The light blue dots denote genes with significant p values but |fold change| $\:\le\:$ 1.2, and the purple dots denote genes that did not meet either the p value or the fold change threshold. **(D-F)** Hierarchical clustering of the top DEGs affected by sex (Bonferroni-corrected *p* < 0.05) in each source. Each row corresponds to one gene and each column corresponds to one sample. **(G)** Top GO pathways enriched by DEGs affected by sex in each genotype. **(H)** Venn diagrams illustrating the overlap of DEGs across different comparisons

To determine the influence of biological sex on gene expression, we analyzed sex-related DEGs and associated pathway enrichment across all three sources of *APOE*-TR mice. This analysis aimed to uncover conserved and source-specific transcriptional signatures linked to sex differences, which may impact neurobiological phenotypes relevant to aging and *APOE*-associated pathologies. We assessed the impact of biological sex on differential gene expression and associated molecular pathways within each source of *APOE*-TR mice. Overall, the number of sex-related DEGs was modest across all three models (Fig. 6A-C, H). Specifically, we identified 17 upregulated and 9 downregulated DEGs in TAC females versus males (Fig. 6A), 14 upregulated and 9 downregulated DEGs in CAF females versus males (Fig. 6B), and 11 upregulated and 12 downregulated DEGs in JAX females versus males (Fig. 6C). Heatmaps of top sex-related DEGs revealed distinct expression profiles (Fig. 6D-F). In TAC mice, *Prss16*, *Prl2c3*, *Prl2c2*, and *Tmem174* were elevated in females, while Pard3bos2 was upregulated in males (Fig. 6D). In CAF mice, *Treml4* and *Tmem174* were more highly expressed in females, whereas *Zfp677* was downregulated (Fig. 6E). In JAX mice, *Trps1*, *Zfp677*, *Ddx4*, and *Tdrd1* were expressed at lower levels in females, while *B4galnt3* and *Scx* showed female-biased upregulation (Fig. 6F). Pathway enrichment analyses revealed that DEGs between sexes across all three sources consistently mapped to demethylation-associated pathways. Notably, only in CAF mice was the Toll-like receptor (TLR) signaling pathway significantly enriched in the female vs. male comparison (Fig. 6G), suggesting potential source-specific immune regulation linked to sex.

### Identification of brain gene co-expression modules associated with source, *APOE* genotype, and sex

**Fig. 7:**
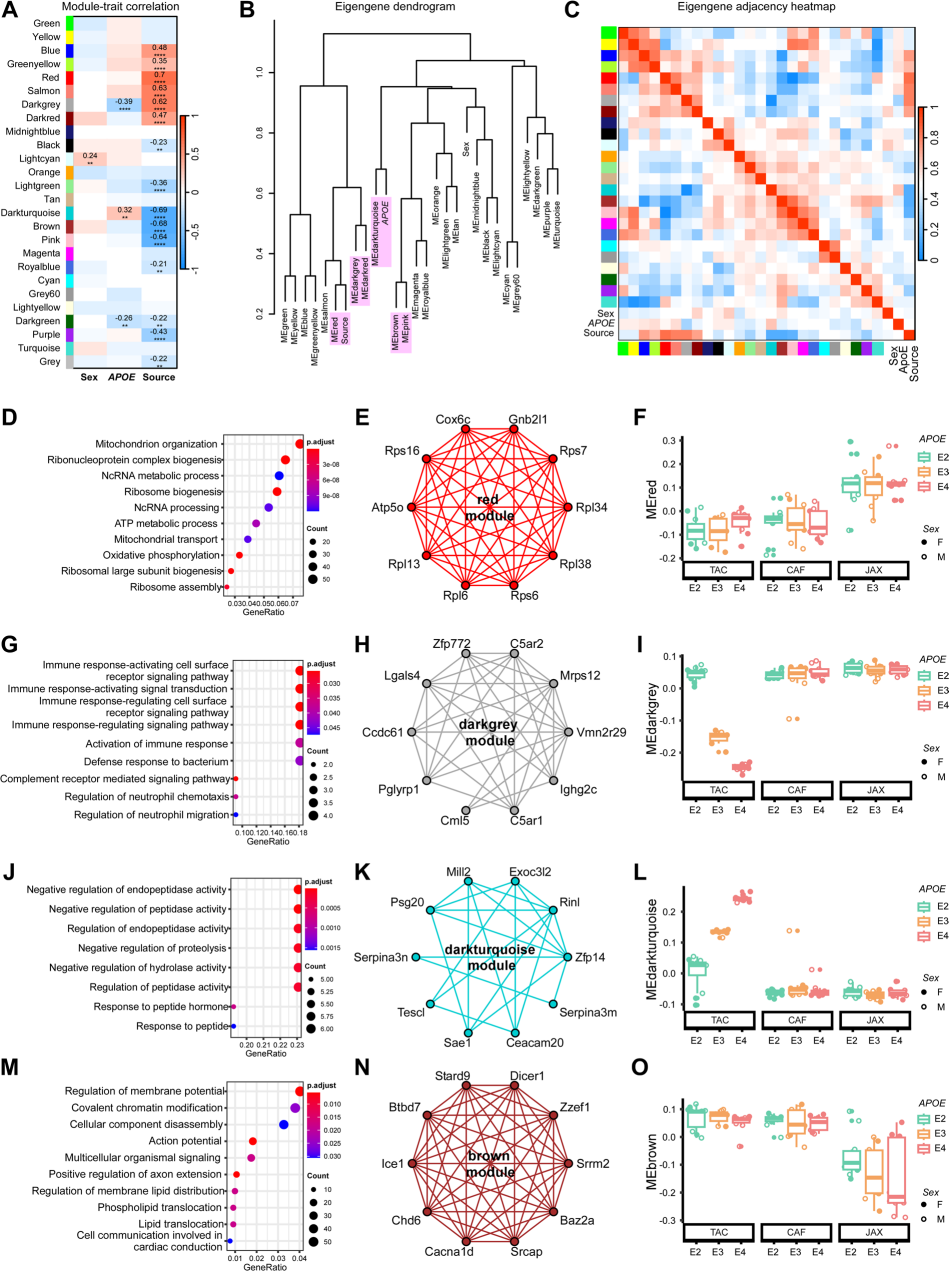
Impact of source, *APOE* genotype, and sex on gene co-expression networks of the mouse brain transcriptomes. **(A)** The correlation between module eigengenes (MEs) and source, *APOE* genotype, and sex. The values in the heatmap are Pearson’s correlation coefficients. Stars represent significant correlations: **p < 0.05; ****p < 0.001. Modules with positive values (red) indicate positive correlation of MEs with source, *APOE4* genotype, or sex; modules with negative values (blue) indicate negative correlation of MEs with these traits. **(B)** The ME network representing the relationships between modules, source, *APOE* genotype, and sex. The y axis shows the dissimilarity of eigengenes. **(C)** Heatmap of the eigengene adjacency matrix. Each row and column correspond to one eigengene (labeled by module color) or a trait of interest. Within the heatmap, red indicates positive correlation and blue indicates negative correlation. **(D**,** G**,** J**,** and M)** Enriched GO pathways in the red, darkgrey, darkturquoise and brown modules. **(E**,** H**,** K and N)** Network plots of the top 10 genes with the highest intramodular connectivity (hub genes) in the red **(E)**, darkgrey **(H)**, darkturquoise **(K)** and brown **(N)** modules. **(F**,** I**,** L and O)** MEs in the red **(F)**, darkgrey **(I)**, darkturquoise **(L)**, and brown **(O)** modules across different source, *APOE* genotype, and sex

To contextualize gene expression changes at a systems level, we performed weighted gene co-expression network analysis (WGCNA) [31, 44] on the bulk brain transcriptomic data. This analysis identified nineteen distinct co-expression modules (Fig. 7A), each representing a group of genes with similar expression patterns. Among these, fifteen modules showed significant associations with mouse source, including six upregulated modules (red, salmon, darkgrey, darkred, blue, and greenyellow) and nine downregulated modules (darkturquoise, brown, pink, purple, lightgreen, black, darkgreen, grey, and royalblue) (Fig. 7A). Additionally, three modules were significantly correlated with *APOE* genotype: darkturquoise (upregulated), and darkgrey and darkgreen (downregulated) (Fig. 7A). Only one module, lightcyan, showed a modest but significant association with sex, exhibiting higher expression levels in females compared to males (Fig. 7A). Further analysis of eigengene-trait relationships confirmed strong correlations between the red module and source, the darkturquoise module and *APOE* genotype, and both the brown and pink modules with source-specific expression differences (Fig. 7B, C). These findings emphasize the impact of mouse model origin and *APOE* genotype on brain-wide transcriptional networks. In addition, to assess the structural robustness of identified gene networks, we evaluated their preservation across subgroups. We found that most modules were highly preserved across mouse source, sex, and *APOE* genotype (Zsummary > 10, Figure S2A-S2H). These results suggest that the identified modules capture broadly shared coexpression patterns rather than being artifacts driven by any single subgroup.

To explore the biological significance of these modules, we performed functional enrichment analyses and identified the top 10 intramodular hub genes for each module. The red module, strongly upregulated in JAX mice, was enriched for mitochondrial transport, ATP metabolism, oxidative phosphorylation, ribosome biogenesis, and non-coding RNA processing (Fig. 7D, F). Its hub genes included those involved in mitochondrial respiration (e.g., *Cox6c*), ATP synthesis (*Atp5o*), and ribosome structure/function (*Rpl6*,* Rpl13*,* Rpl34*,* Rpl38*,* Rps6*,* Rps7*,* Rps16*, and *Gnb2l1*) (Fig. 7E). The darkgrey module, also significantly associated with source, was upregulated in CAF and JAX mice and showed a strong *APOE* genotype-dependent pattern in TAC mice (*APOE2* > *APOE3* > *APOE4*) (Fig. 7I). It was enriched for immune response pathways (Fig. 7G) and included hub genes directly (*Lgals4*,* C5ar1*,* C5ar2*,* Zfp772*,* Ighg2c*,* Pglyrp1*) and indirectly (*Mrps12*,* Ccdc61*,* Vmn2r29*,* Cml5*) linked to immune function (Fig. 7H). In contrast, the darkturquoise module was downregulated in CAF and JAX mice but exhibited a reciprocal isoform-dependent expression in TAC (*APOE2* < *APOE3* < *APOE4*) (Fig. 7L). This module was enriched for endopeptidase regulatory processes (Fig. 7J), with hub genes including *Serpina3n*,* Serpina3m*,* Tescl*,* Sae1*,* Mill2*,* Rinl*,* Exoc3l2*,* Zfp14*,* Ceacam20*, and *Psg20* (Fig. 7K). The brown module was downregulated in JAX mice and showed *APOE* genotype-dependent expression (*APOE2* > *APOE3* ≈ *APOE4*) (Fig. 7O). Genes in this module were enriched in pathways related to chromatin modification and cellular disassembly, with key players such as *Dicer1*,* Chd6*,* Baz2a*,* Stard9*,* Btbd7*,* Srcap*, and *Zzef1* involved in chromatin regulation and lipid translocation (Fig. 7M, N). The pink module, highly correlated with the brown module and downregulated in JAX mice, was similarly enriched for chromatin modification pathways (Figure S3K, S3L). The module expression profiles of blue, greenyellow, darkred, and black were modestly elevated in JAX mice. These modules were enriched for biological processes such as mitochondrial respiration and RNA metabolism (blue), RNA processing and protein folding (greenyellow), protein phosphorylation and muscle development (darkred), and brain development and cell polarity (black) (Figure S3A-S3H). In contrast, the lightgreen, darkgreen, and purple modules were slightly downregulated in JAX mice and were associated with fatty acid and compound metabolism (lightgreen), muscle system processes (darkgreen), and pathways involved in synapse organization, dendritic development, and neurotransmitter activity (purple) (Figure S3I, S3J; S3M-S3P). Although the salmon and royalblue modules were significantly associated with source, no enriched pathways were identified (Figure S3S, S3T). 

We next examined gene co-expression modules associated with *APOE* genotype. Among the 19 modules identified, the darkgrey, darkturquoise, and darkgreen modules were significantly correlated with *APOE* genotype. The darkturquoise module was upregulated, whereas the darkgrey and darkgreen modules were downregulated with respect to *APOE *genotype (Fig. 7A). As previously noted, the darkgrey module was enriched for immune-related pathways. Notably, its module eigengene (ME) demonstrated a clear *APOE* genotype-dependent expression pattern (*APOE2* > *APOE3* > *APOE4*) in TAC mice, but this trend was not observed in CAF or JAX mice. The darkturquoise module was enriched for genes involved in the regulation of endopeptidase activity (Fig. 7J), and its ME displayed the opposite *APOE* genotype trend (*APOE2* < *APOE3* < *APOE4*) in TAC mice only (Fig. 7L). The darkgreen module also showed genotype-specific differences, but these were modest and only evident in JAX mice (Figure S3M, S3N). Further, in JAX *APOE*-TR mice, the MEs of several modules, including blue, greenyellow, darkred, black, lightgreen, pink, darkgreen, purple, salmon, and royalblue, exhibited isoform-dependent differences and were enriched for distinct biological pathways. Specifically, the blue and greenyellow modules were enriched for pathways related to mitochondrial respiration, RNA processing, and protein folding (Figure S3A-S3D). The darkred module was associated with protein phosphorylation, kinase activity, and muscle development (Figure S3E, S3F). The black module was enriched for processes including hindbrain development, cerebellar cortex morphogenesis, centriole assembly, and replication (Figure S3G, S3H). The purple module included genes involved in synaptic organization, dendritic development and morphogenesis, and neurotransmitter activity (Figure S3O, Figure S3P). Notably, all of these modules were downregulated in *APOE2* mice but upregulated in *APOE3* and *APOE4* mice. In contrast, the lightgreen module exhibited the opposite trend, with higher expression in *APOE2* mice and lower levels in *APOE3* and *APOE4* mice. Genes in this module were enriched for fatty acid metabolism and drug metabolic processes (Figure S3I, S3J). Together, these results highlight genotype-specific transcriptional differences in JAX *APOE*-TR mice and underscore the importance of considering *APOE* genotype when interpreting functional outcomes and selecting models for mechanistic studies. These findings suggest that the expression of these modules is influenced by both *APOE* genotype and the source of the mouse models. The lightcyan module, significantly associated with sex (Fig. 7A), was modestly elevated in female mice compared to males (Figure S3R). Genes in this module were enriched for developmental processes, including pattern specification, skeletal morphogenesis, and nervous system development (Figure S3Q).

To reinforce the transcriptomic conclusions, we performed biological validation through western blotting to measure the protein level of Serpina3n, a member of the Serpin family of proteins that primarily functions as a serine protease inhibitor, playing crucial roles in inflammation and various diseases regulating endopeptidase regulatory processes. It is one of the hub genes in darkturquoise module (Fig. 7K) and one of the top DEGs affected by *APOE* genotype in TAC (Fig. 5J). We found that the protein levels of Serpina3N increased significantly in TAC *APOE4* mice compared to those in TAC *APOE2* and *APOE3* mice (Figure S6A, S6D), consistent with the transcriptomic findings. However, this isoform-dependent difference was not observed in CAF and JAX mice (Figure S6B, S6C, S6E, S6F), which is also consistent with the RNA sequencing results.

## Discussion

Humanized *APOE*-TR mice are indispensable tools for dissecting the isoform-specific roles of apoE in AD and related neurodegenerative disorders [23–25, 40, 45, 46]. However, the inherent diversity in their genetic engineering strategies and sources has raised concerns regarding the comparability and reproducibility of findings across studies. Our comprehensive molecular characterization of three widely used *APOE*-TR models, sourced from Taconic Biosciences (TAC), Cure Alzheimer’s Fund (CAF), and Jackson Laboratory (JAX), reveals substantial and critical variability in apoE levels, peripheral lipid profiles, and brain transcriptomic landscapes.

Different targeting strategies and the resulting alleles (e.g., inclusion of the NeoR cassette in the TAC mice, loxP allele in the CAF mice, and CRISPR-mediated gene editing used in JAX mice) can lead to variable gene expression levels [47–50], even when encoding the same *APOE* gene. Consistent with this, we observed source-dependent differences in apoE protein levels across the brain, liver, and plasma, suggesting that the underlying targeting strategy and associated regulatory elements may influence expression efficiency. Such differences underscore the critical need for direct comparisons of *APOE* genotype mouse models from the same source, particularly in studies investigating dose-sensitive phenotypes such as inflammation [51]. We noticed that our findings on brain *APOE* amount and gene expression signatures differ somewhat from previous reports [25, 40, 42, 43]. This discrepancy may be attributable to differences in sample preparation; our study utilized pulverized whole mouse hemispheres which could potentially mask brain region-specific effects, whereas prior studies primarily analyzed dissected cortex or hippocampus tissues. Such variation in brain region sampling may contribute to the observed differences in apoE amount patterns and brain transcriptomic signatures. These differences may also be influenced by genetic background. For example, the CAF *APOE*-TR mice studied in this work were on a C57BL/6N background whereas the same mice studied by Hyunh, et al. [25] had been backcrossed from a C57BL/6N background to a C57BL/6J background. Furthermore, the observed differences in apoE protein levels were mirrored by distinct plasma lipid profiles, including LDL, HDL, triglycerides, and total cholesterol, which are critical readouts in *APOE*-related metabolic and neurodegenerative disease studies [52–55]. Given that apoE plays a central role in lipid transport and homeostasis [3, 7, 18], these findings emphasize the importance of considering model-specific lipid phenotypes when interpreting experimental outcomes. The three commonly used humanized *APOE*-TR mouse models - TAC, CAF, and JAX - each offer distinct advantages and limitations. TAC mice, as an early-established model, reliably recapitulate human *APOE* isoform effects in vivo but retain NeoR selection cassette, which may interfere with endogenous regulatory elements and affect expression fidelity. In contrast, CAF mice feature excised selection markers and incorporate loxP sites flanking the human *APOE* sequence, enabling both conventional expression and Cre-mediated conditional deletion of the *APOE* gene allele in a cell type-specific manner. As such, these mice offer greater experimental flexibility and cleaner genomic integration; however, this model has not been widely used compared to the TAC mice. JAX mice are the most recent *APOE*-TR model generated to serve the broader scientific community, using a targeting strategy with excised selection markers. Notably, the *APOE2* and *APOE3* mice were isogenic lines generated by CRISPR editing of *APOE4* mice; however, the JAX mice do not offer the option of conditional *APOE* deletion with the *APOE2* and *APOE3* mice have potential, albeit minimal, risk of off-target effects from CRISPR editing. Therefore, choice of model should align with experimental goals: TAC mice remain useful with well-characterized phenotypes, CAF mice provide the versatility of both conventional expression and conditional deletion, and JAX mice are created for the broader scientific community. Due to different substrain genetic backgrounds (C57BL/6N for CAF and TAC models, and C57BL/6J for JAX models), the CAF and TAC models would match to the IMPC knockout mouse models being created on B6N, while the JAX models on B6J match to most of the widely used AD models, including all of the new models being created by the NIA-funded MODEL-AD consortium. Knock-in models of *APOE* protective variants have also been created in the JAX models: the Christchurch (R154S) was made in both *APOE3* and *APOE4*, and the Jacksonville (V236E) variant in *APOE3*. The *APOE* Christchurch (R154S) variant was also generated in the *APOE3* CAF line [56]. Another practical consideration is that TAC mice have breeding restrictions, CAF mice are not commercially available but can be obtained through an MTA via Cure Alzheimer’s fund, and JAX mice are commercially available for the broader scientific community without breeding or usage restrictions.

In addition to apoE protein levels and plasma lipid profiles, we also characterized brain gene expression characteristics of the *APOE*-TR mice from the three sources. WGCNA [31] revealed that “source” exerts the greatest influence on global brain gene expression, surpassing *APOE* genotype or sex. These source-specific effects likely reflect differences in genetic constructs (e.g., targeting strategies, residual selection cassettes, floxed alleles), genetic substrain, potential mutations introduced through CRISPR gene editing, or epigenetic alterations during model development. Additionally, variations in apoE isoform levels both within and across different sources can also contribute to source-dependent brain transcriptomic profiles. Further, distinct sets of DEGs and enriched pathways were observed when comparing *APOE* genotypes both within and across sources, indicating that biological conclusions drawn from one line may not readily generalize to others. Our results clearly indicate that brain transcriptomic profiles in JAX mice differ markedly from those in TAC and CAF mice, whereas TAC and CAF exhibit greater overall similarity. Specifically, most module eigengenes (MEs) (red, brown, blue, greenyellow, black, lightgreen, pink, darkgreen, purple, salmon, and royalblue) showed distinct levels in JAX mice compared to TAC and CAF mice, aligning with our PCA results that demonstrate clear separation of JAX mice from TAC and CAF mice. Interestingly, despite these global differences, sex-related DEGs in JAX mice were enriched for the same demethylation-related pathways as those observed in TAC and CAF mice. Pathway enrichment analyses revealed a large number of overlapping pathways in TAC vs. JAX and CAF vs. JAX comparisons, while only a few pathways were enriched in TAC vs. CAF, consistent with the smaller number of DEGs identified between TAC and CAF. Additionally, DEGs among *APOE* genotypes in JAX mice differed from those in TAC and CAF mice, resulting in distinct sets of enriched pathways by *APOE* genotype. Collectively, our findings indicate that JAX mice exhibit a distinct brain transcriptomic profile, which is at least partially attributable to differences between the C57BL/6J and C57BL/6N genetic backgrounds [57, 58]; the overlap between DEGs influenced by source (TAC vs. JAX or CAF vs. JAX) and those associated with genetic background (C57BL/6N vs. C57BL/6J) also supported this (Figure S4A-S4F, Table S3). Interestingly, only in TAC mice did *APOE* genotype robustly influence the expression of immune- and peptide-related modules in a clear isoform-dependent manner: darkgrey (immune response-related, Fig. 7G-I) showed *APOE2* > *APOE3* > *APOE4*, while the darkturquoise (peptide activity-associated, Fig. 7J-L) exhibited the opposite trend of *APOE2* < *APOE3* < *APOE4*. These findings align with previous reports highlighting apoE isoforms as key regulators of the innate immune response in the brain and their critical role in neuroinflammation and related neurodegeneration [16, 59, 60]. Notably, no significant DEGs were detected among *APOE* genotypes in CAF mouse brains, and ME expression levels were comparable across genotypes, indicating a high degree of transcriptomic similarity among *APOE* genotypes in this particular model, at least in the brains of young adult mice. The lack of overlap in *APOE* genotype comparisons across sources is primarily due to the CAF cohort, where DEG analyses for *APOE* genotype did not yield any significant DEGs under our thresholds. As a result, no overlap was detected when all three sources were compared in the Venn diagram; however, a small number of overlapping DEGs were identified between TAC and JAX (Fig. 5M). While this overlap is minimal, it suggests limited but detectable consistency between specific sources.

Importantly, we detected sex-specific transcriptional signatures and pathway enrichments-particularly in demethylation, highlighting the need to account for sex as a biological variable [61, 62], even in young adult animals without pathology. We found that separation of sex was clear in TAC and CAF, but less so in JAX. To confirm sex identity in JAX mice, we performed hierarchical clustering using X and Y chromosome genes, which clearly separated samples by sex (Figure S5A-S5C), ruling out any sample mix-up. Notably, sex-specific DEGs of those *APOE*-TR mice share common enrichment in the demethylation-related pathways, suggesting an active shift in the brain’s epigenetic landscape. Interestingly, DNA methylation and demethylation are known to influence sex-specific development and potentially play a role in disease pathogenesis [63, 64]. Previous findings indicate sex effects on AD progression, including brain region-specific tau deposition [65], cognitive decline [66], amyloidosis [67], and metabolic processes [68]; especially in the presence of *APOE4* genotype. It is worth noting that even in the absence of pathology and at a young age (3–4 months), all three sources of *APOE*-TR mice already shared enrichment in demethylation-related pathways, indicating minimal source differences in this respect.

## Conclusion

To our knowledge, this is the first comprehensive study directly comparing humanized *APOE*-TR mouse models from all three major sources: Taconic Biosciences (TAC), Cure Alzheimer’s Fund (CAF), and the The Jackson Laboratory (JAX). By systematically evaluating these models, we provide critical insights into molecular differences that can potentially inform future research on *APOE* genotype effects and their functional implications under aging and disease conditions.

### Limitation and future directions

Our study, while comprehensive, has several limitations. First, although the animals were age- and background-matched, subtle differences in substrain, housing or breeding conditions between sources may contribute to phenotypic variation. Second, we focused on transcriptomic and metabolic characterization in a baseline, non-pathological state; the differential responses of these models under AD-relevant stressors (e.g., aging, amyloidosis, or tauopathy) remains to be elucidated. Third, transcriptomics profiling was limited to the brain, and future studies should expand to include comparison of cell type-specific expression.

We hope this study provides critical information to guide future research on human *APOE* isoforms using animal models. Given the significant differences in apoE isoform levels, lipid profiles, and gene expression among different sources of *APOE*-TR mice, we recommend that each lab try to consistently use a single, specific source. This will ensure the consistency and comparability of different studies. However, if the goal is to study the role of cell type-specific effect of *APOE* with *cre*-dependent gene ablation, the CAF mice are the only model that can be utilized. For collaborative studies or those within a research consortium, we also suggest selecting a single source of *APOE*-TR mice to facilitate cross-referencing of study outcomes. If a lab must change its source of *APOE*-TR mice, we urge caution when drawing conclusions across studies and recommend validation of key findings. Ultimately, all study outcomes should be compared to or validated against findings in humans or human samples. We hope other research groups will continue to compare *APOE*-TR mice from different sources under various conditions, including physiological aging and in the presence of AD pathologies.

Moving forward, the application of single-cell transcriptomics and spatial omics will enable deeper resolution of *APOE* genotype effects within distinct brain cell populations. Functional studies combining these models with disease associated models may also help delineate how apoE isoforms interact with disease pathologies. Importantly, the field would significantly benefit from harmonized guidelines for humanized *APOE* model selection and reporting to improve reproducibility and cross-study comparability. Our findings lay the groundwork for such standardization and establishes a framework for this transition, demonstrating that rigorous, head-to-head molecular characterization is vital for translatable preclinical research.

## Supplementary Information

Below is the link to the electronic supplementary material.

Supplementary Material 1

Supplementary Material 2

Supplementary Material 3

Supplementary Material 4

## Data Availability

Bulk RNA-seq data have been deposited at Synapse database with accession number syn69774864 (https://doi.org/10.7303/syn69774864) and are available as of the date of publication. Visualization of brain gene expression of this work are available in the “EPAAD” website ([Gene Expresssion Database | EPAAD.org] (https://www.epaad.org/gene-expresssion-database)) as of the date of publication. Further data and code supporting the findings of this study are available from the corresponding authors on request.

## Abbreviations

TR: Targeted-replacement
apoE: Apolipoprotein E
AD: Alzheimer’s disease
TAC: Taconic Biosciences
CAF: Cure Alzheimer’s Fund
JAX: The Jackson Laboratory
LDL: Low-density lipoprotein
HDL: High-density lipoprotein
DEGs: Differentially expressed genes
WGCNA: Weighted gene co-expression network analysis
LC-MS/MS: Liquid chromatography–tandem mass spectrometry
NCE: Normalized collision energy
PVCA: Principal variance component analysis

## References

[1] Hanlon CS, Rubinsztein DC. Arginine residues at codons 112 and 158 in the apolipoprotein E gene correspond to the ancestral state in humans. Atherosclerosis. 1995;112:85–90. 7772071 10.1016/0021-9150(94)05402-5

[2] Verghese PB, Castellano JM, Garai K, Wang Y, Jiang H, Shah A, Bu G, Frieden C, Holtzman DM. ApoE influences amyloid-beta (Abeta) clearance despite minimal apoE/Abeta association in physiological conditions. Proc Natl Acad Sci U S A. 2013;110:E1807–1816. 23620513 10.1073/pnas.1220484110 PMC3651443

[3] Liu CC, Liu CC, Kanekiyo T, Xu H, Bu G. Apolipoprotein E and Alzheimer disease: risk, mechanisms and therapy. Nat Rev Neurol. 2013;9:106–18. 23296339 10.1038/nrneurol.2012.263 PMC3726719

[4] Huang YA, Zhou B, Wernig M, Sudhof TC. ApoE2, ApoE3, and ApoE4 Differentially Stimulate APP Transcription and Abeta Secretion. Cell. 2017;168:427–e441421. 28111074 10.1016/j.cell.2016.12.044 PMC5310835

[5] Yamazaki Y, Zhao N, Caulfield TR, Liu CC, Bu G. Apolipoprotein E and Alzheimer disease: pathobiology and targeting strategies. Nat Rev Neurol. 2019;15:501–18. 31367008 10.1038/s41582-019-0228-7 PMC7055192

[6] Raulin AC, Doss SV, Trottier ZA, Ikezu TC, Bu G, Liu CC. ApoE in Alzheimer’s disease: pathophysiology and therapeutic strategies. Mol neurodegeneration. 2022;17:72. 10.1186/s13024-022-00574-4 PMC9644639 36348357

[7] Blumenfeld J, Yip O, Kim MJ, Huang Y. Cell type-specific roles of APOE4 in Alzheimer disease. Nat Rev Neurosci. 2024;25:91–110. 38191720 10.1038/s41583-023-00776-9 PMC11073858

[8] Chen Y, Strickland MR, Soranno A, Holtzman DM. Apolipoprotein E: Structural Insights and Links to Alzheimer Disease Pathogenesis. Neuron. 2021;109:205–21. 33176118 10.1016/j.neuron.2020.10.008 PMC7931158

[9] Martens YA, Zhao N, Liu CC, Kanekiyo T, Yang AJ, Goate AM, Holtzman DM, Bu G. ApoE Cascade Hypothesis in the pathogenesis of Alzheimer’s disease and related dementias. Neuron. 2022;110:1304–17. 35298921 10.1016/j.neuron.2022.03.004 PMC9035117

[10] DeTure MA, Dickson DW. The neuropathological diagnosis of Alzheimer’s disease. Mol neurodegeneration. 2019;14:32. 10.1186/s13024-019-0333-5 PMC6679484 31375134

[11] Liu CC, Wang N, Chen Y, Inoue Y, Shue F, Ren Y, Wang M, Qiao W, Ikezu TC, Li Z, et al. Cell-autonomous effects of APOE4 in restricting microglial response in brain homeostasis and Alzheimer’s disease. Nat Immunol. 2023;24:1854–66. 37857825 10.1038/s41590-023-01640-9 PMC11980647

[12] Wang Z, Zhang L, Qin C. Alzheimer’s disease pathogenesis: standing at the crossroad of lipid metabolism and immune response. Mol Neurodegener. 2025;20:67. 40468377 10.1186/s13024-025-00857-6 PMC12139291

[13] Thal DR, Poesen K, Vandenberghe R, De Meyer S. Alzheimer’s disease neuropathology and its estimation with fluid and imaging biomarkers. Mol neurodegeneration. 2025;20:33. 10.1186/s13024-025-00819-y PMC11907863 40087672

[14] Belaidi AA, Bush AI, Ayton S. Apolipoprotein E in Alzheimer’s disease: molecular insights and therapeutic opportunities. Mol neurodegeneration. 2025;20:47. 10.1186/s13024-025-00843-y PMC12023563 40275327

[15] Yang LG, March ZM, Stephenson RA, Narayan PS. Apolipoprotein E in lipid metabolism and neurodegenerative disease. Trends Endocrinol Metab. 2023;34:430–45. 37357100 10.1016/j.tem.2023.05.002 PMC10365028

[16] Murphy KB, Hu D, Wolfs L, Rohde SK, Fauro GL, Geric I, Mancuso R, De Strooper B, Marzi SJ. The APOE isoforms differentially shape the transcriptomic and epigenomic landscapes of human microglia xenografted into a mouse model of Alzheimer’s disease. Nat Commun. 2025;16:4883. 40419479 10.1038/s41467-025-60099-4 PMC12106835

[17] Wang C, Xiong M, Gratuze M, Bao X, Shi Y, Andhey PS, Manis M, Schroeder C, Yin Z, Madore C, et al. Selective removal of astrocytic APOE4 strongly protects against tau-mediated neurodegeneration and decreases synaptic phagocytosis by microglia. Neuron. 2021;109:1657–e16741657. 33831349 10.1016/j.neuron.2021.03.024 PMC8141024

[18] Wang N, Wang M, Jeevaratnam S, Rosenberg C, Ikezu TC, Shue F, Doss SV, Alnobani A, Martens YA, Wren M, et al. Opposing effects of apoE2 and apoE4 on microglial activation and lipid metabolism in response to demyelination. Mol neurodegeneration. 2022;17:75. 10.1186/s13024-022-00577-1 PMC9682675 36419137

[19] Wang N, Cai L, Pei X, Lin Z, Huang L, Liang C, Wei M, Shao L, Guo T, Huang F, et al. Microglial apolipoprotein E particles contribute to neuronal senescence and synaptotoxicity. iScience. 2024;27:110006. 38868202 10.1016/j.isci.2024.110006 PMC11167441

[20] Maloney B, Ge YW, Alley GM, Lahiri DK. Important differences between human and mouse APOE gene promoters: limitation of mouse APOE model in studying Alzheimer’s disease. J Neurochem. 2007;103:1237–57. 17854398 10.1111/j.1471-4159.2007.04831.x

[21] Liao F, Zhang TJ, Jiang H, Lefton KB, Robinson GO, Vassar R, Sullivan PM, Holtzman DM. Murine versus human apolipoprotein E4: differential facilitation of and co-localization in cerebral amyloid angiopathy and amyloid plaques in APP transgenic mouse models. Acta Neuropathol Commun. 2015;3:70. 26556230 10.1186/s40478-015-0250-y PMC4641345

[22] Fagan AM, Watson M, Parsadanian M, Bales KR, Paul SM, Holtzman DM. Human and murine ApoE markedly alters A beta metabolism before and after plaque formation in a mouse model of Alzheimer’s disease. Neurobiol Dis. 2002;9:305–18. 11950276 10.1006/nbdi.2002.0483

[23] Jankowsky JL, Zheng H. Practical considerations for choosing a mouse model of Alzheimer’s disease. Mol neurodegeneration. 2017;12:89. 10.1186/s13024-017-0231-7 PMC5741956 29273078

[24] Sullivan PM, Mezdour H, Aratani Y, Knouff C, Najib J, Reddick RL, Quarfordt SH, Maeda N. Targeted replacement of the mouse apolipoprotein E gene with the common human APOE3 allele enhances diet-induced hypercholesterolemia and atherosclerosis. J Biol Chem. 1997;272:17972–80. 9218423 10.1074/jbc.272.29.17972

[25] Huynh TV, Wang C, Tran AC, Tabor GT, Mahan TE, Francis CM, Finn MB, Spellman R, Manis M, Tanzi RE, et al. Lack of hepatic apoE does not influence early Abeta deposition: observations from a new APOE knock-in model. Mol neurodegeneration. 2019;14:37. 10.1186/s13024-019-0337-1 PMC6796484 31623648

[26] Ritchie ME, Phipson B, Wu D, Hu Y, Law CW, Shi W. Smyth GK: limma powers differential expression analyses for RNA-sequencing and microarray studies. Nucleic Acids Res. 2015;43:e47. 25605792 10.1093/nar/gkv007 PMC4402510

[27] Hoffman GE, Schadt EE. variancePartition: interpreting drivers of variation in complex gene expression studies. BMC Bioinformatics. 2016;17:483. 27884101 10.1186/s12859-016-1323-z PMC5123296

[28] Worakul T, Laplaza R, Das S, Wodrich MD, Corminboeuf C. Microkinetic Molecular Volcano Plots for Enhanced Catalyst Selectivity and Activity Predictions. ACS Catal. 2024;14:9829–39. 38988648 10.1021/acscatal.4c01175 PMC11232097

[29] Gao CH, Yu G, Cai P. ggVennDiagram: An Intuitive, Easy-to-Use, and Highly Customizable R Package to Generate Venn Diagram. Front Genet. 2021;12:706907. 34557218 10.3389/fgene.2021.706907 PMC8452859

[30] Gu Z, Eils R, Schlesner M. Complex heatmaps reveal patterns and correlations in multidimensional genomic data. Bioinformatics. 2016;32:2847–9. 27207943 10.1093/bioinformatics/btw313

[31] Langfelder P, Horvath S. WGCNA: an R package for weighted correlation network analysis. BMC Bioinformatics. 2008;9:559. 19114008 10.1186/1471-2105-9-559 PMC2631488

[32] Wang Z, Kavdia K, Dey KK, Pagala VR, Kodali K, Liu D, Lee DG, Sun H, Chepyala SR, Cho JH, et al. High-throughput and Deep-proteome Profiling by 16-plex Tandem Mass Tag Labeling Coupled with Two-dimensional Chromatography and Mass Spectrometry. Journal of visualized experiments: JoVE; 2020. 10.3791/61684 PMC7752892 32894271

[33] Hughes CS, Moggridge S, Muller T, Sorensen PH, Morin GB, Krijgsveld J. Single-pot, solid-phase-enhanced sample preparation for proteomics experiments. Nat Protoc. 2019;14:68–85. 30464214 10.1038/s41596-018-0082-x

[34] Cheng D, Hoogenraad CC, Rush J, Ramm E, Schlager MA, Duong DM, Xu P, Wijayawardana SR, Hanfelt J, Nakagawa T, et al. Relative and absolute quantification of postsynaptic density proteome isolated from rat forebrain and cerebellum. Mol Cell proteomics: MCP. 2006;5:1158–70. 16507876 10.1074/mcp.D500009-MCP200

[35] Yarbro JM, Shrestha HK, Wang Z, Zhang X, Zaman M, Chu M, Wang X, Yu G, Peng J. Proteomic landscape of Alzheimer’s disease: emerging technologies, advances and insights (2021–2025). Molecular neurodegeneration 2025, 20:83. 10.1186/s13024-025-00874-5 PMC12257826 40660303

[36] Niu M, Cho JH, Kodali K, Pagala V, High AA, Wang H, Wu Z, Li Y, Bi W, Zhang H, et al. Extensive Peptide Fractionation and y(1) Ion-Based Interference Detection Method for Enabling Accurate Quantification by Isobaric Labeling and Mass Spectrometry. Anal Chem. 2017;89:2956–63. 28194965 10.1021/acs.analchem.6b04415 PMC5467445

[37] Sullivan PM, Mezdour H, Quarfordt SH, Maeda N. Type III hyperlipoproteinemia and spontaneous atherosclerosis in mice resulting from gene replacement of mouse Apoe with human Apoe*2. J Clin Invest. 1998;102:130–5. 9649566 10.1172/JCI2673 PMC509074

[38] Knouff C, Hinsdale ME, Mezdour H, Altenburg MK, Watanabe M, Quarfordt SH, Sullivan PM, Maeda N. Apo E structure determines VLDL clearance and atherosclerosis risk in mice. J Clin Invest. 1999;103:1579–86. 10359567 10.1172/JCI6172 PMC408371

[39] Mahan TE, Wang C, Bao X, Choudhury A, Ulrich JD, Holtzman DM. Selective reduction of astrocyte apoE3 and apoE4 strongly reduces Abeta accumulation and plaque-related pathology in a mouse model of amyloidosis. Mol neurodegeneration. 2022;17:13. 10.1186/s13024-022-00516-0 PMC8811969 35109920

[40] Foley KE, Hewes AA, Garceau DT, Kotredes KP, Carter GW, Sasner M, Howell GR. The APOE (epsilon3/epsilon4) Genotype Drives Distinct Gene Signatures in the Cortex of Young Mice. Front Aging Neurosci. 2022;14:838436. 35370604 10.3389/fnagi.2022.838436 PMC8967347

[41] McLean JW, Bhattrai A, Vitali F, Raikes AC, Wiegand JL, Brinton RD. Contributions of sex and genotype to exploratory behavior differences in an aged humanized APOE mouse model of late-onset Alzheimer’s disease. Learn Mem. 2022;29:321–31. 36206387 10.1101/lm.053588.122 PMC9488030

[42] Sepulveda J, Luo N, Nelson M, Ng CAS, Rebeck GW. Independent APOE4 knock-in mouse models display reduced brain APOE protein, altered neuroinflammation, and simplification of dendritic spines. J Neurochem. 2022;163:247–59. 35838553 10.1111/jnc.15665 PMC9613529

[43] Zhao N, Ren Y, Yamazaki Y, Qiao W, Li F, Felton LM, Mahmoudiandehkordi S, Kueider-Paisley A, Sonoustoun B, Arnold M, et al. Alzheimer’s Risk Factors Age, APOE Genotype, and Sex Drive Distinct Molecular Pathways. Neuron. 2020;106:727–e742726. 32199103 10.1016/j.neuron.2020.02.034 PMC7388065

[44] Liu Y. CWGCNA: an R package to perform causal inference from the WGCNA framework. NAR genomics Bioinf. 2024;6:lqae042. 10.1093/nargab/lqae042 PMC11044439 38666214

[45] Williams T, Borchelt DR, Chakrabarty P. Therapeutic approaches targeting Apolipoprotein E function in Alzheimer’s disease. Mol neurodegeneration. 2020;15:8. 10.1186/s13024-020-0358-9 PMC6995170 32005122

[46] Balu D, Karstens AJ, Loukenas E, Maldonado Weng J, York JM, Valencia-Olvera AC, LaDu MJ. The role of APOE in transgenic mouse models of AD. Neurosci Lett. 2019;707:134285. 31150730 10.1016/j.neulet.2019.134285 PMC6717006

[47] Stifter SA, Greter M. STOP floxing around: Specificity and leakiness of inducible Cre/loxP systems. Eur J Immunol. 2020;50:338–41. 32125704 10.1002/eji.202048546

[48] Li S, Xia L. Precise gene replacement in plants through CRISPR/Cas genome editing technology: current status and future perspectives. aBIOTECH. 2020;1:58–73. 36305005 10.1007/s42994-019-00009-7 PMC9590512

[49] Hans S, Zoller D, Hammer J, Stucke J, Spiess S, Kesavan G, Kroehne V, Eguiguren JS, Ezhkova D, Petzold A, et al. Cre-Controlled CRISPR mutagenesis provides fast and easy conditional gene inactivation in zebrafish. Nat Commun. 2021;12:1125. 33602923 10.1038/s41467-021-21427-6 PMC7893016

[50] Sander JD, Joung JK. CRISPR-Cas systems for editing, regulating and targeting genomes. Nat Biotechnol. 2014;32:347–55. 24584096 10.1038/nbt.2842 PMC4022601

[51] Tzioras M, Davies C, Newman A, Jackson R, Spires-Jones T. Invited Review: APOE at the interface of inflammation, neurodegeneration and pathological protein spread in Alzheimer’s disease. Neuropathol Appl Neurobiol. 2019;45:327–46. 30394574 10.1111/nan.12529 PMC6563457

[52] Dunk MM, Li J, Liu S, Casanova R, Chen JC, Espeland MA, Hayden KM, Manson JE, Rapp SR, Shadyab AH, et al. Associations of dietary cholesterol and fat, blood lipids, and risk for dementia in older women vary by APOE genotype. Alzheimer’s Dement J Alzheimer’s Assoc. 2023;19:5742–54. 10.1002/alz.13358 PMC10784407 37438877

[53] Johnson LA, Olsen RH, Merkens LS, DeBarber A, Steiner RD, Sullivan PM, Maeda N, Raber J. Apolipoprotein E-low density lipoprotein receptor interaction affects spatial memory retention and brain ApoE levels in an isoform-dependent manner. Neurobiol Dis. 2014;64:150–62. 24412220 10.1016/j.nbd.2013.12.016 PMC3936477

[54] Qi Y, Liu J, Wang W, Wang M, Zhao F, Sun J, Liu J, Zhao D. Apolipoprotein E-containing high-density lipoprotein (HDL) modifies the impact of cholesterol-overloaded HDL on incident coronary heart disease risk: A community-based cohort study. J Clin Lipidol. 2018;12:89–e9882. 29217413 10.1016/j.jacl.2017.11.003

[55] Kypreos KE, van Dijk KW, van Der Zee A, Havekes LM, Zannis VI. Domains of apolipoprotein E contributing to triglyceride and cholesterol homeostasis in vivo. Carboxyl-terminal region 203–299 promotes hepatic very low density lipoprotein-triglyceride secretion. J Biol Chem. 2001;276:19778–86. 11279066 10.1074/jbc.M100418200

[56] Chen Y, Song S, Parhizkar S, Lord J, Zhu Y, Strickland MR, Wang C, Park J, Tabor GT, Jiang H, et al. APOE3ch alters microglial response and suppresses Abeta-induced tau seeding and spread. Cell. 2024;187:428–e445420. 38086389 10.1016/j.cell.2023.11.029 PMC10842861

[57] Mekada K, Yoshiki A. Substrains matter in phenotyping of C57BL/6 mice. Exp Anim. 2021;70:145–60. 33441510 10.1538/expanim.20-0158 PMC8150240

[58] Nemoto S, Kubota T, Ohno H. Metabolic differences and differentially expressed genes between C57BL/6J and C57BL/6 N mice substrains. PLoS ONE. 2022;17:e0271651. 36548271 10.1371/journal.pone.0271651 PMC9778930

[59] Keene CD, Cudaback E, Li X, Montine KS, Montine TJ. Apolipoprotein E isoforms and regulation of the innate immune response in brain of patients with Alzheimer’s disease. Curr Opin Neurobiol. 2011;21:920–8. 21907569 10.1016/j.conb.2011.08.002 PMC3237894

[60] Kloske CM, Barnum CJ, Batista AF, Bradshaw EM, Brickman AM, Bu G, Dennison J, Gearon MD, Goate AM, Haass C, et al. APOE and immunity: Research highlights. Alzheimer’s Dement J Alzheimer’s Assoc. 2023;19:2677–96. 10.1002/alz.13020 PMC13158998 36975090

[61] Giorgio J, Jonson C, Wang Y, Yokoyama JS, Wang J, Jagust WJ. Alzheimer’s Disease Neuroimaging I: Variable and interactive effects of Sex, APOE epsilon4 and TREM2 on the deposition of tau in entorhinal and neocortical regions. Nat Commun. 2025;16:5812. 40595476 10.1038/s41467-025-60370-8 PMC12214702

[62] Gamache J, Yun Y, Chiba-Falek O. Sex-dependent effect of APOE on Alzheimer’s disease and other age-related neurodegenerative disorders. Disease models & mechanisms; 2020. p. 13. 10.1242/dmm.045211 PMC7473656 32859588

[63] Cortes LR, Forger NG. DNA methylation and demethylation shape sexual differentiation of neurochemical phenotype. Horm Behav. 2023;151:105349. 37001316 10.1016/j.yhbeh.2023.105349 PMC10133097

[64] Cisternas CD, Cortes LR, Bruggeman EC, Yao B, Forger NG. Developmental changes and sex differences in DNA methylation and demethylation in hypothalamic regions of the mouse brain. Epigenetics. 2020;15:72–84. 31378140 10.1080/15592294.2019.1649528 PMC6961693

[65] Yan S, Zheng C, Paranjpe MD, Li Y, Li W, Wang X, Benzinger TLS, Lu J, Zhou Y. Sex modifies APOE epsilon4 dose effect on brain tau deposition in cognitively impaired individuals. Brain. 2021;144:3201–11. 33876815 10.1093/brain/awab160 PMC8634082

[66] Petersen KK, Grober E, Lipton RB, Sperling RA, Buckley RF, Aisen PS, Ezzati A. Impact of sex and APOE epsilon4 on the association of cognition and hippocampal volume in clinically normal, amyloid positive adults. Alzheimer’s Dement. 2022;14:e12271. 10.1002/dad2.12271 PMC8828988 35155730

[67] Nemes S, Logan PE, Manchella MK, Mundada NS, La Joie R, Polsinelli AJ, Hammers DB, Koeppe RA, Foroud TM, Nudelman KN, et al. Sex and APOE epsilon4 carrier effects on atrophy, amyloid PET, and tau PET burden in early-onset Alzheimer’s disease. Alzheimer’s Dement J Alzheimer’s Assoc. 2023;19(Suppl 9):S49–63. 10.1002/alz.13403 PMC10811272 37496307

[68] Arnold M, Nho K, Kueider-Paisley A, Massaro T, Huynh K, Brauner B, MahmoudianDehkordi S, Louie G, Moseley MA, Thompson JW, et al. Sex and APOE epsilon4 genotype modify the Alzheimer’s disease serum metabolome. Nat Commun. 2020;11:1148. 32123170 10.1038/s41467-020-14959-w PMC7052223

