# Supplementary material for: Molecular characterization of humanized APOE mouse models reveals source and genotype dependent differences
Source: Mol Neurodegener. 2026 Mar 24;21:26. doi: 10.1186/s13024-026-00940-6 (PMC13185305; doi:10.1186/s13024-026-00940-6)
Supplement: Supplementary file 1 — Supplementary Material 1 [file 13024_2026_940_MOESM1_ESM.docx]

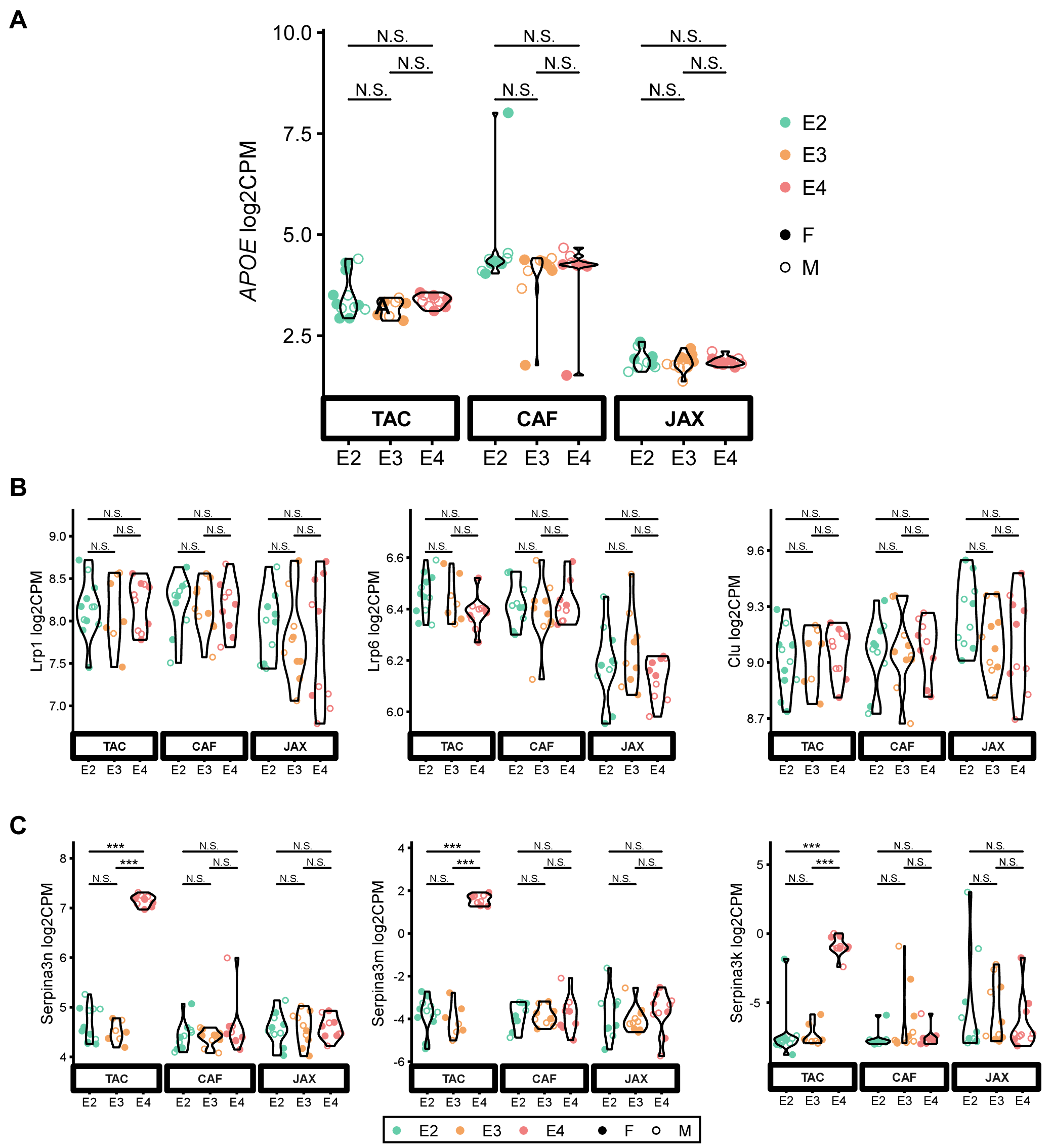


**Figure S1.** **Expression of *APOE* and key related genes among three sources of *APOE*-TR mice. (A)** Human *APOE* genotype-specific expression determined by RNA sequencing. **(B)** Transcriptional expression of *Lrp1*, *Lrp6*, and *Clu* determined by RNA sequencing. **(C)** Transcriptional expression of *Serpina3n, Serpina3m, Serpina3k* determined by RNA sequencing.


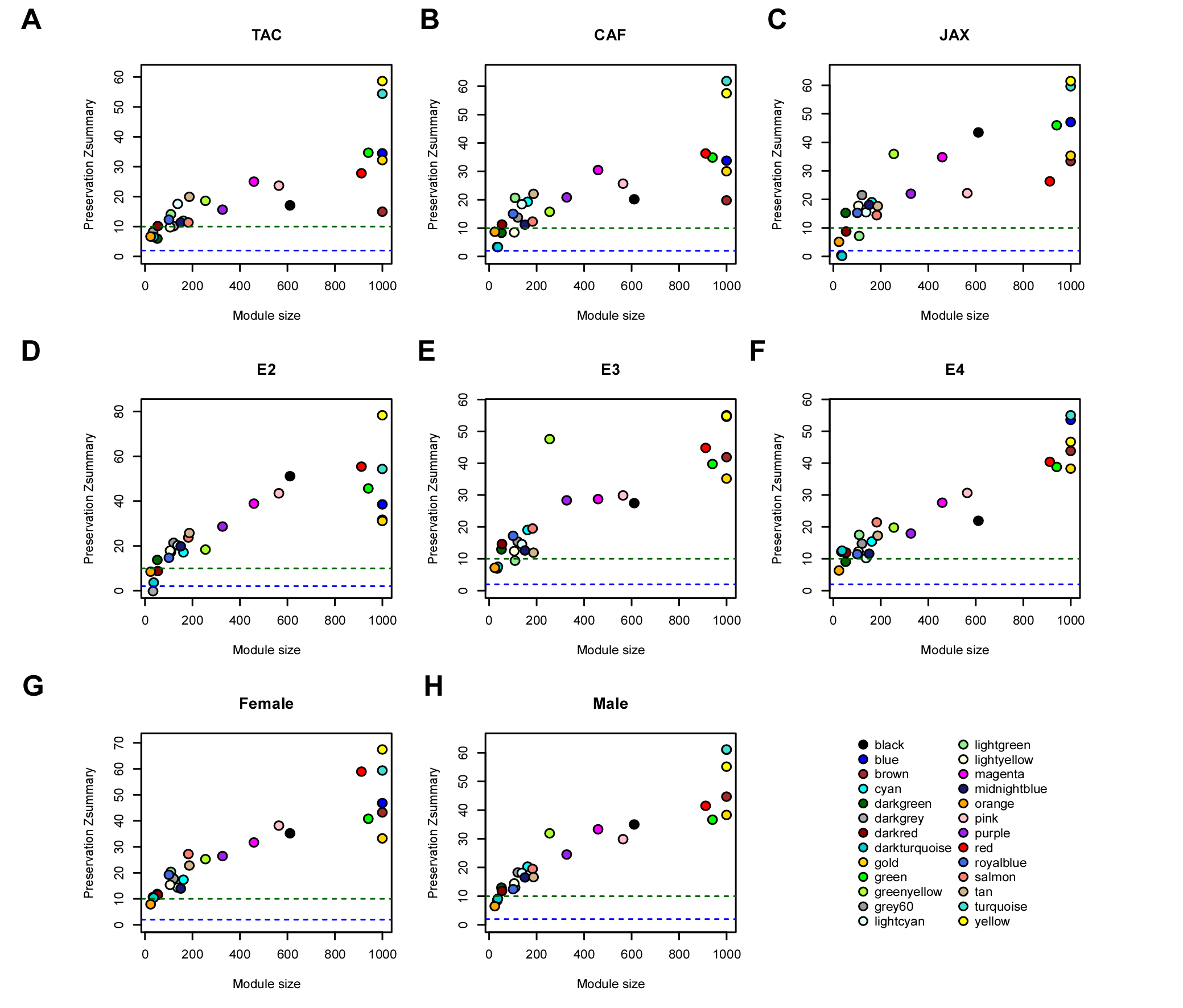


**Figure S2.** **Module preservation analysis across *APOE* genotype, source, and sex.** **(A-C)** Preservation of modules in Taconic (TAC), Cure Alzheimer's Fund (CAF), and Jackson Laboratory (JAX) mice. Most modules show strong preservation (Zsummary > 10).  **(D-F)** Comparison of network stability across APOE2, APOE3, and APOE4 mice. Most modules exhibit high preservation (Zsummary > 10). **(G, H)** Module preservation between female and male cohorts, showing consistent network architecture across sexes. Each point represents a discrete co-expression module, plotted by its size (number of genes) against its preservation score.


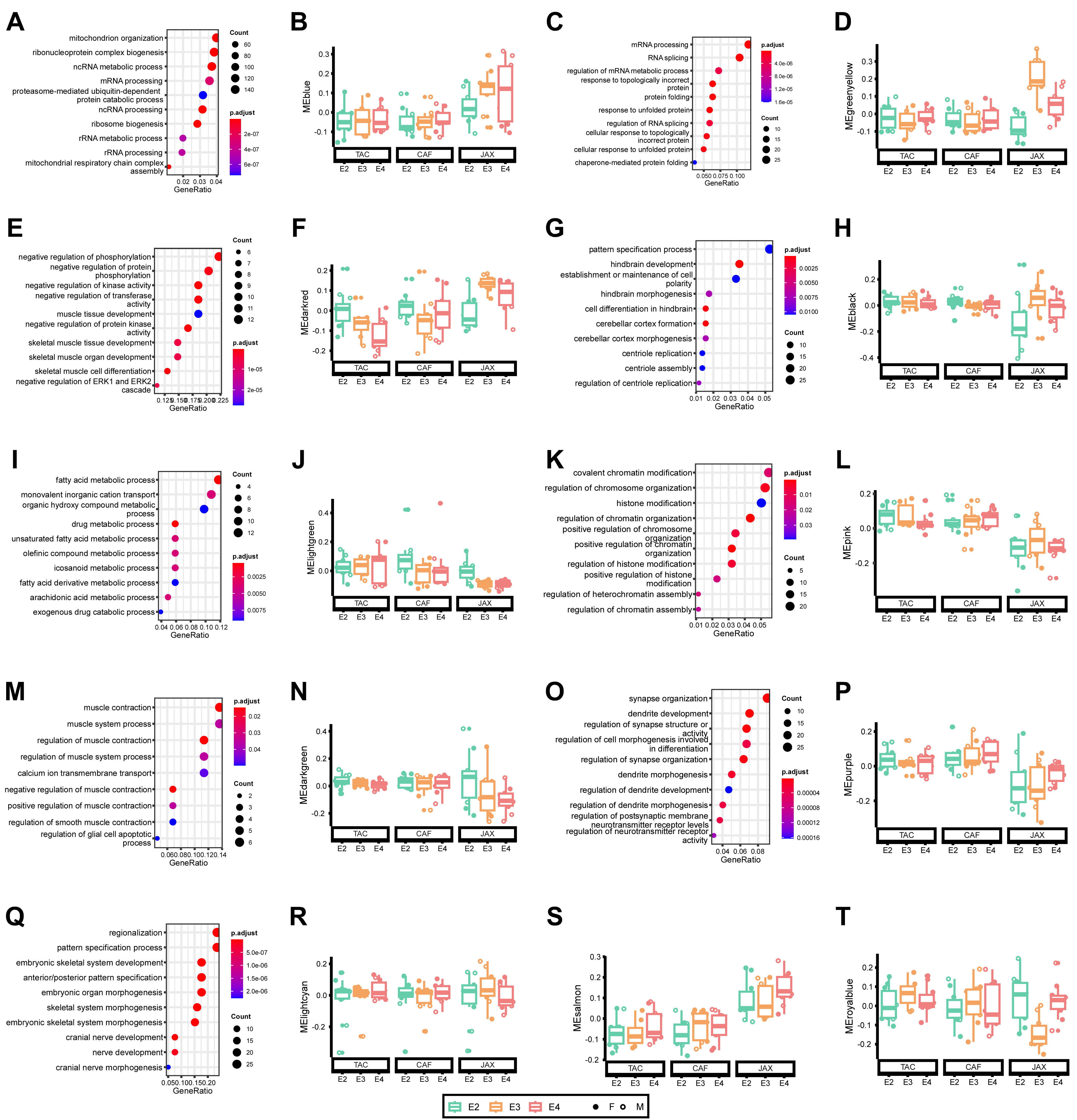


**Figure S3.** **Gene co-expression networks associated with source, *APOE* genotype, and sex.** **(A, C, E, G, I, K, M, O, Q)** Top pathways enriched in blue module **(A)**, greenyellow module **(C)**, darkred module **(E)**, black module **(G)**, lightgreen module **(I)**, pink module **(K)**, darkgreen module **(M)**, purple module **(O)**, and lightcyan module **(Q)**.  **(B, D, F, H, J, L, N, P, R, S, T)** MEs of blue **(B)**, greeyellow **(D)**, darkred **(F)**, black **(H)**, lightgreen **(J)**, pink **(L)**, darkgreen **(N)**, purple **(P)**, lightcyan **(R)**, salmon **(S)**, and royalblue **(T)** modules across different sources, *APOE* genotype, and sex (n=3-7 mice/source/genotype/sex). The upper and lower lines in the boxplots represent the maximum and minimum values after Tukey’s test. The center line represents the median.


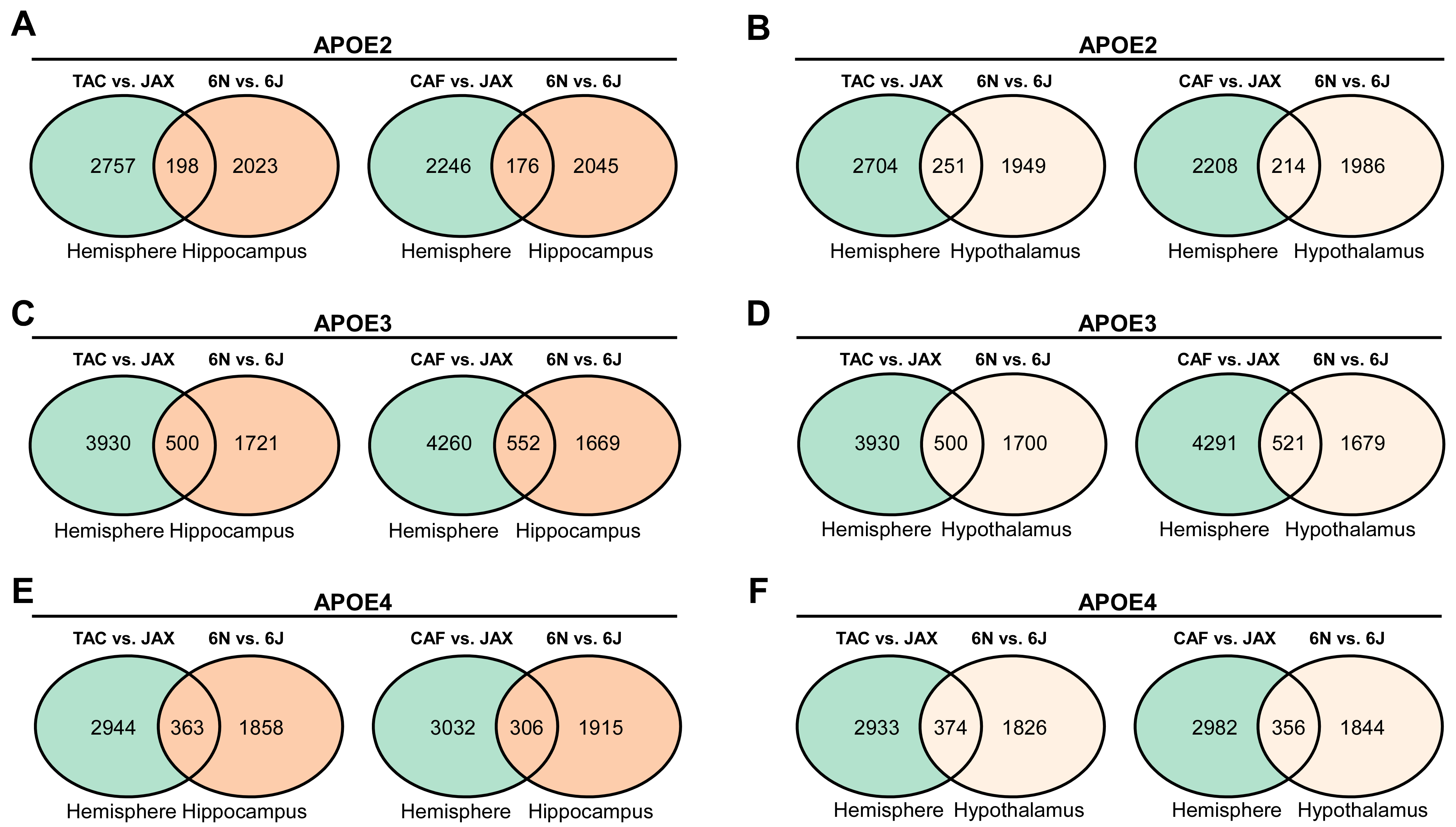


**Figure S4. Comparation of DEGs influenced by source and genetic background. (A, B)** Venn diagrams illustrate the number of overlapping DEGs between TAC vs. JAX, or CAF vs. JAX (pulverized hemisphere) and C57BL6N vs. C57BL6J in hippocampus or hypothalamus in APOE2 group. **(C, D)** Venn diagrams illustrate the number of overlapping DEGs between TAC vs. JAX, or CAF vs. JAX (pulverized hemisphere) and C57BL6N vs. C57BL6J in hippocampus or hypothalamus in APOE3 group. **(E, F)** Venn diagrams illustrate the number of overlapping DEGs between TAC vs. JAX, or CAF vs. JAX (pulverized hemisphere) and C57BL6N vs. C57BL6J in hippocampus or hypothalamus in APOE4 group.


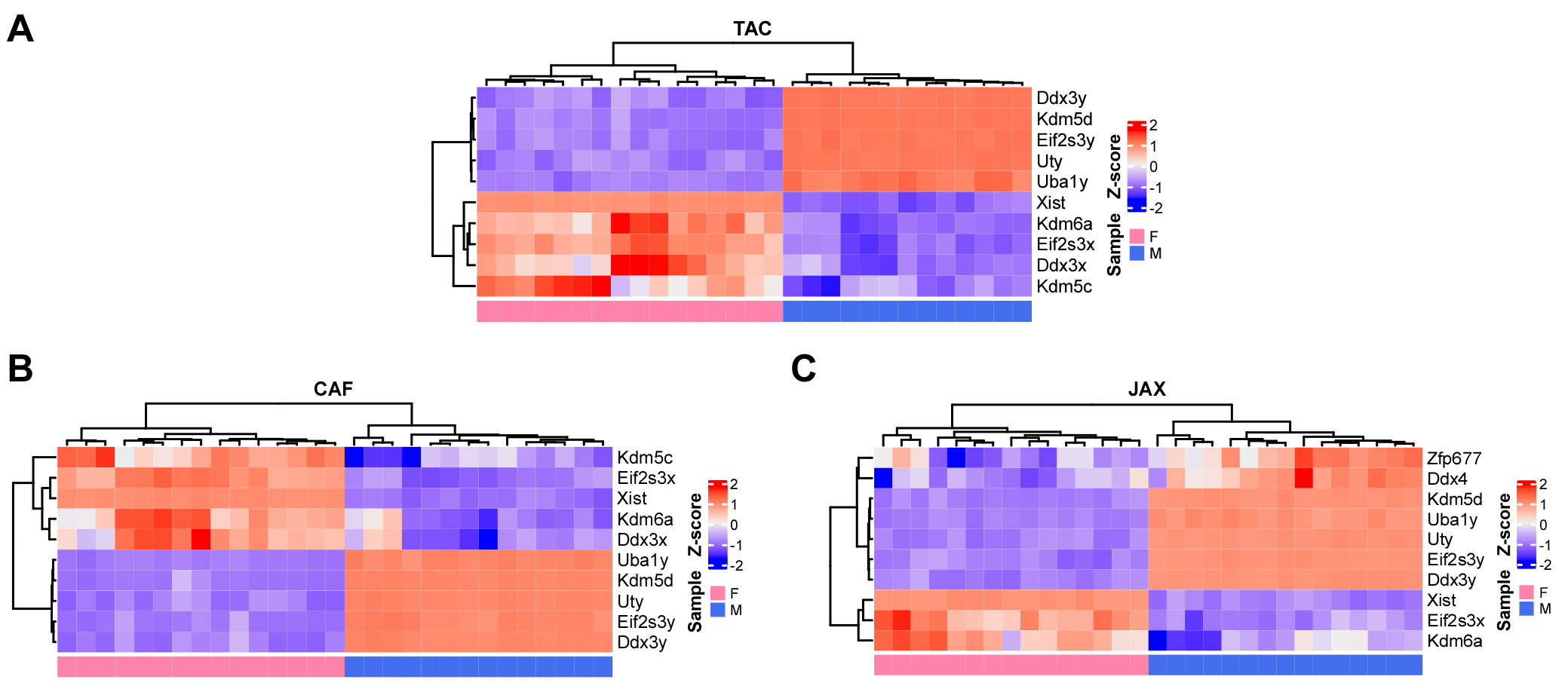


**Figure S5.** **Hierarchical clustering of the top DEGs affected by sex
(with genes on X or Y chromosome). (A)** Top DEGs affected by sex among TAC mice. **(B)** Top DEGs affected by sex among CAF mice. **(C)** Top DEGs affected by sex among JAX mice.


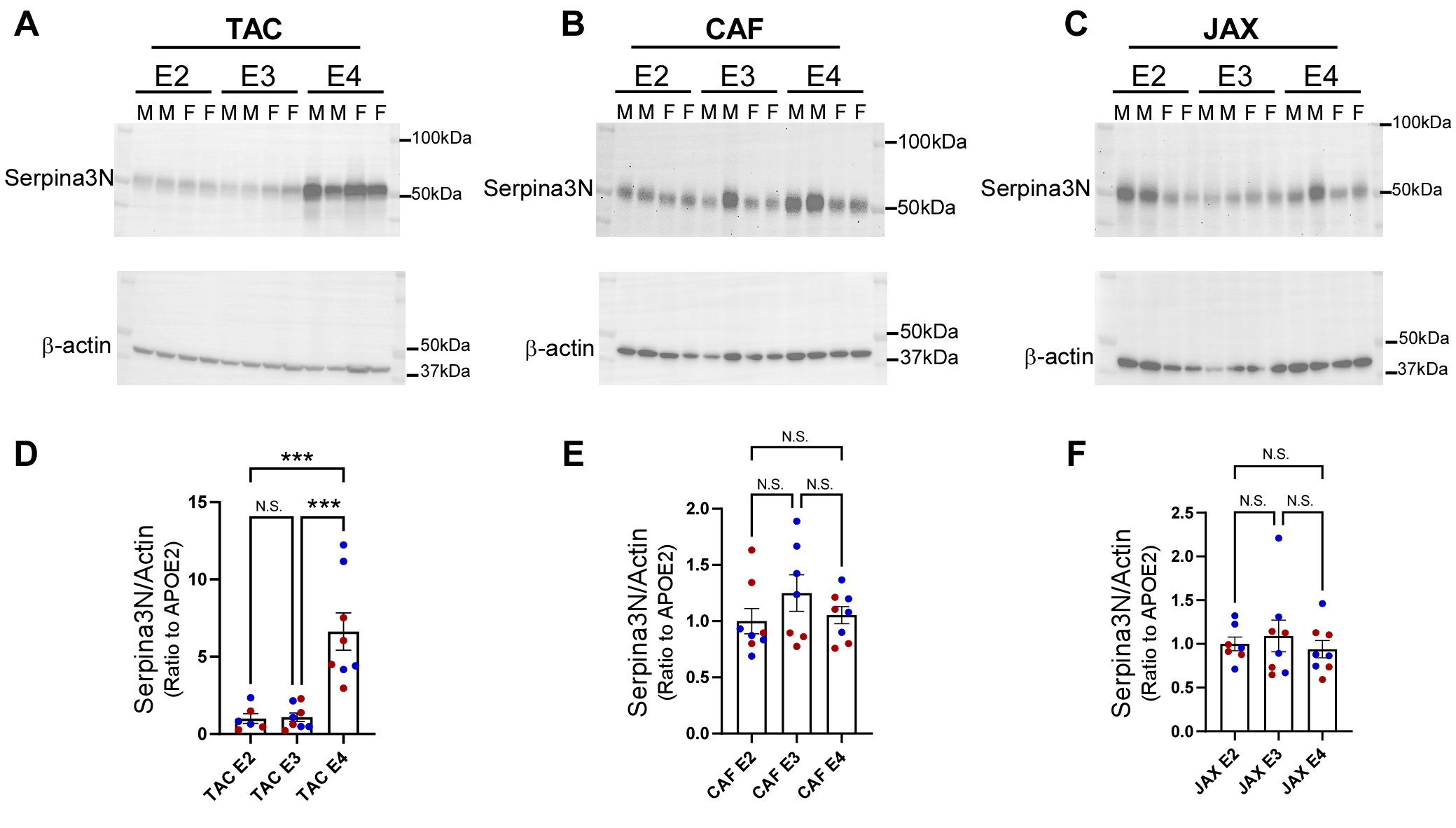


**Figure S6.** **Validation of Serpina3N protein amount in *APOE*-TR mice from three sources. (A-C)** Representative images of Serpina3N expression in the brains of *APOE*-TR mice. **(D-F)** Quantification of Serpina3N expression. n=3-4 mice per genotype/sex/source. One-way ANOVA with Tukey multiple comparisons test was used for comparison of *APOE* genotype. Data are presented as mean ± SEM. ns, no significant. Significance was defined as ***p < 0.001.
